# Faricimab 6 mg Versus Aflibercept 8 mg in Treatment-Naïve Neovascular Age-Related Macular Degeneration: A Protocol-Standardised In Silico Study

**DOI:** 10.3390/ph19081316

**Published:** 2026-08-20

**Authors:** Georgios D. Panos

**Affiliations:** 1 Division of Ophthalmology and Visual Sciences, School of Medicine, University of Nottingham, Nottingham NG7 2UH, UK; gdpanos@gmail.com; Tel.: +30-231-330-3110; 2 Department of Ophthalmology, AHEPA University Hospital, School of Medicine, Aristotle University of Thessaloniki, 54636 Thessaloniki, Greece

**Keywords:** aflibercept 8 mg, faricimab, neovascular age-related macular degeneration, in silico study, virtual eyes, microsimulation, treatment burden, treat-and-extend, evidence synthesis

## Abstract

**Background**: Faricimab 6 mg and aflibercept 8 mg permit extended treatment intervals in neovascular age-related macular degeneration (nAMD), but their pivotal programmes used different loading and maintenance schedules. This study compared treatment burden under the same loading and treat-and-extend protocol and included an exploratory longitudinal analysis of published aggregate visual and anatomical outcomes. **Methods**: Treatment-specific Dirichlet distributions represented persistent Q8W, Q12W and Q16W capability. Both treatments received injections at weeks 0, 4, 8 and 16, followed by identical four-week extensions from Q8W to Q16W. The primary analysis comprised 30,000 evidence draws, 100,000 paired virtual eyes, a 1.5-million-pair scenario grid and a nested five-million-pair probabilistic analysis. Exploratory multilevel meta-regressions estimated BCVA and change in retinal thickness through week 52; IRF, SRF and complete retinal dryness were analysed separately. **Results**: Mean injections with faricimab and aflibercept 8 mg were 7.255 and 7.212 at week 52 and 11.522 and 11.144 at week 104. The nested 104-week difference was −0.393 injection (95% uncertainty interval −0.682 to −0.107), but broad source-weight uncertainty included no difference and credible source analyses changed the direction of the contrast. Exploratory week-52 BCVA gains were 5.65 and 6.29 letters, respectively; the difference was 0.64 letter (95% uncertainty interval −1.41 to 2.70) and should be interpreted in the context of three aflibercept 8 mg study families. The expanded retinal-thickness sensitivity model estimated changes of −171.8 and −147.2 micrometres, while the between-treatment direction varied in the three-dose-only analysis. Complete retinal dryness was projected in 57.0% and 69.0% of eyes, respectively, based on three study families per treatment. **Conclusions**: Use of the same protocol predicted essentially equal one-year burden and a small two-year fixed-weight difference that varied across source choices. The exploratory longitudinal projections, informed by three independent aflibercept 8 mg study families at one year, provide supportive context for the visual and anatomical findings but should be interpreted as hypothesis-generating rather than confirmatory.

## 1. Introduction

Intravitreal inhibition of vascular endothelial growth factor (VEGF) has transformed the prognosis of neovascular age-related macular degeneration (nAMD), but sustained disease control often requires repeated monitoring and injections [1,2]. The resulting burden affects patients, caregivers, clinicians and treatment capacity, and undertreatment in routine practice can erode the visual gains achieved during initial therapy [3,4]. Recent retinal vascular studies have similarly assessed injection exposure together with visual and anatomical outcomes, supporting an integrated interpretation of treatment frequency and clinical response [5]. Newer agents have therefore been developed not only to preserve visual and anatomical efficacy, but also to extend treatment intervals [6,7].

Faricimab (Vabysmo; Roche Registration GmbH, Grenzach-Wyhlen, Germany) is a bispecific antibody that inhibits VEGF-A and angiopoietin-2. In the TENAYA and LUCERNE trials, faricimab administered after four monthly loading injections and then at intervals up to every 16 weeks produced visual outcomes non-inferior to aflibercept 2 mg every 8 weeks [6]. A personalised treat-and-extend strategy maintained visual and anatomical outcomes through two years [8]. Aflibercept 8 mg (Eylea 8 mg; Bayer AG, Leverkusen, Germany) increases the delivered molar dose of aflibercept and was evaluated in PULSAR after three monthly injections, followed by Q12W or Q16W schedules with protocol-defined interval modification. Visual outcomes were non-inferior to aflibercept 2 mg at week 48 and were maintained through week 96, with some eyes assigned intervals beyond Q16W in year 2 [7,9].

Direct comparison of observed injection counts between these programmes is problematic because the protocols were not aligned. TENAYA/LUCERNE included an additional loading injection and a different disease-activity assessment structure, whereas PULSAR began extended fixed intervals after three monthly injections and permitted protocol-specific modification. Existing network meta-analyses and economic models have generally retained the original trial regimens and have consequently estimated fewer injections with aflibercept 8 mg than with faricimab treat-and-extend [10,11]. Such analyses are clinically informative, but they cannot separate molecular durability from protocol-induced treatment burden.

Contemporary observational evidence also permits a more direct assessment of a three-dose faricimab strategy. Three-dose cohorts have reported functional improvement, substantial central retinal thickness reduction, high rates of retinal fluid resolution and clinically relevant post-loading durability [12,13]. A small direct comparison found similar one-year functional and anatomical outcomes after three versus four faricimab loading injections [14]. More recently, a direct observational comparison of 100 treatment-naïve eyes found comparable BCVA improvement, central retinal thickness reduction and complete fluid resolution after three loading injections of faricimab or aflibercept 8 mg [15]. These data support the plausibility of a common loading phase, although they do not provide long-term randomised evidence under the same maintenance algorithm.

In silico trials have been proposed as a framework for integrating clinical evidence and examining treatment strategies that have not been compared directly under a common protocol [16]. A protocol-standardised probabilistic in silico analysis was therefore developed in which faricimab 6 mg and aflibercept 8 mg received the same three-dose loading phase and the same Q8W-to-Q16W extension protocol. The primary purpose was not to infer visual or anatomical superiority, but to estimate treatment burden conditional on clinically acceptable visual control. The analysis examined whether the apparent injection advantage reported by cross-trial comparisons persisted after removal of the main protocol differences and after allowing for uncertainty in the evidence sources.

## 2. Results

### 2.1. Supporting Visual and Anatomical Evidence

The three-dose faricimab evidence indicated a pooled loading gain of 5.11 ETDRS letters, a mean central retinal thickness reduction of 156.5 micrometres and complete central fluid control in approximately 79.9% of eyes. PULSAR reported week-48 gains of 6.7 and 6.2 letters and retinal thickness reductions of 142 and 147 micrometres for the Q12W and Q16W aflibercept 8 mg groups, respectively [7]. At week 96, visual gains remained 5.6 and 5.5 letters, with retinal thickness reductions of 143.9 and 153.4 micrometres [9].

In the direct observational three-dose comparison, aflibercept 8 mg and faricimab both improved BCVA and reduced retinal thickness, with complete retinal fluid resolution in 80.4% and 87.8% of eyes, respectively; none of the between-treatment differences was statistically significant [15]. These findings were consistent with using visual and anatomical outcomes as an external plausibility guardrail. They did not, however, provide long-term comparative outcomes under the common maintenance protocol.

### 2.2. Evidence-Weighted Capability Distributions

**Figure 1:**
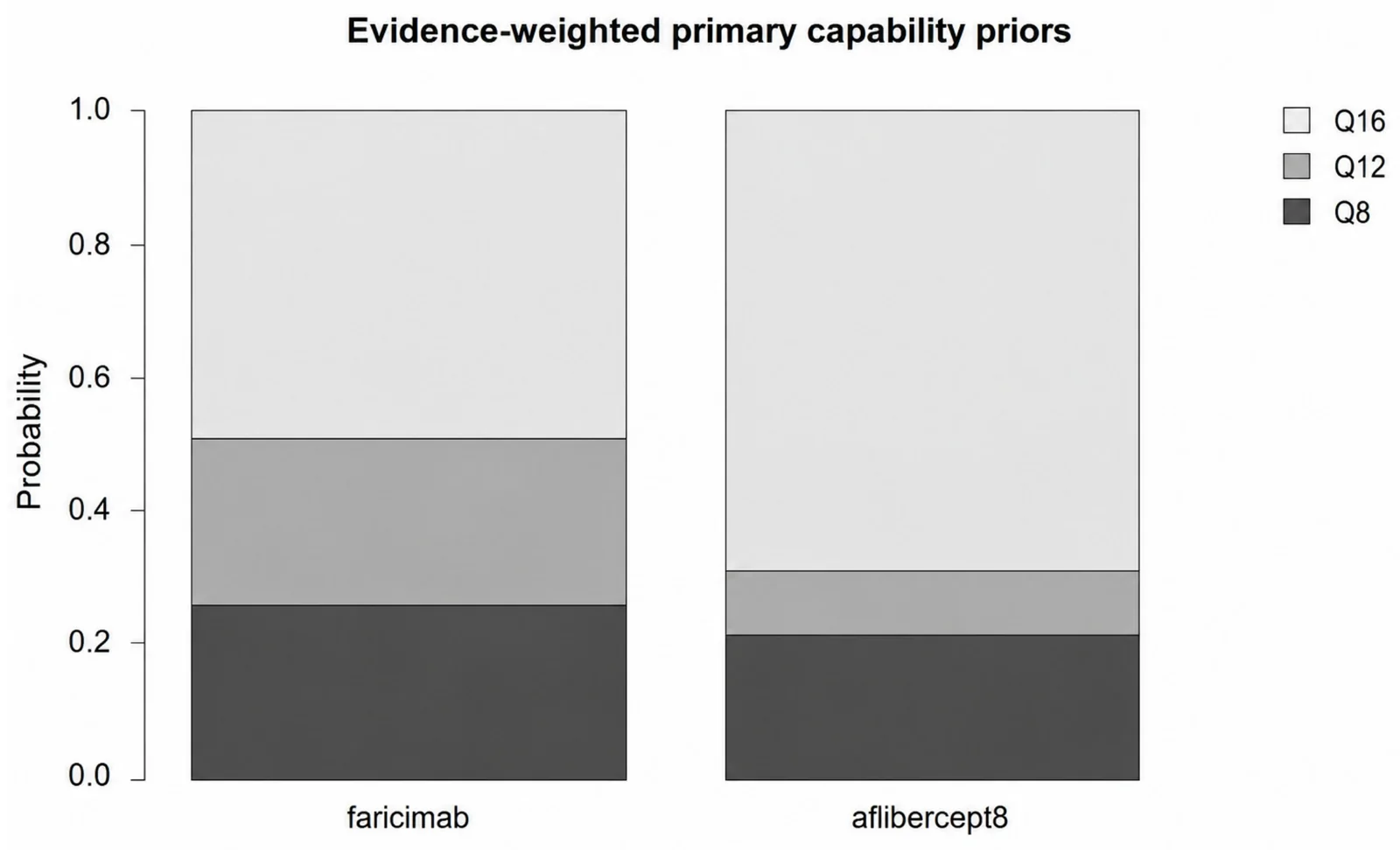
Evidence-weighted Q8W, Q12W and Q16W interval-capability distributions used in the primary analysis. The bars show the mean probability assigned to each persistent maximum interval class after draw-wise evidence synthesis.

The primary faricimab capability distribution was Q8W 25.7% (95% UI 21.3–30.4%), Q12W 24.5% (20.6–28.8%) and Q16W 49.8% (44.6–54.9%). The corresponding aflibercept 8 mg distribution was Q8W 21.1% (17.4–25.1%), Q12W 8.6% (6.0–11.6%) and Q16W 70.3% (65.8–74.6%). Thus, the primary synthesis assigned aflibercept 8 mg a 20.6% higher probability of Q16W capability, while faricimab had a larger intermediate Q12W subgroup (Table 1; Figure 1).

### 2.3. Primary Treatment-Burden Results

At week 52, expected injections were 7.26 with faricimab and 7.21 with aflibercept 8 mg, giving a difference of −0.05 injection (95% UI −0.11 to 0.01). The absolute difference was below 0.5 injection in all uncertainty draws. Expected monitoring visits were 8.26 and 8.21, respectively.

**Figure 2:**
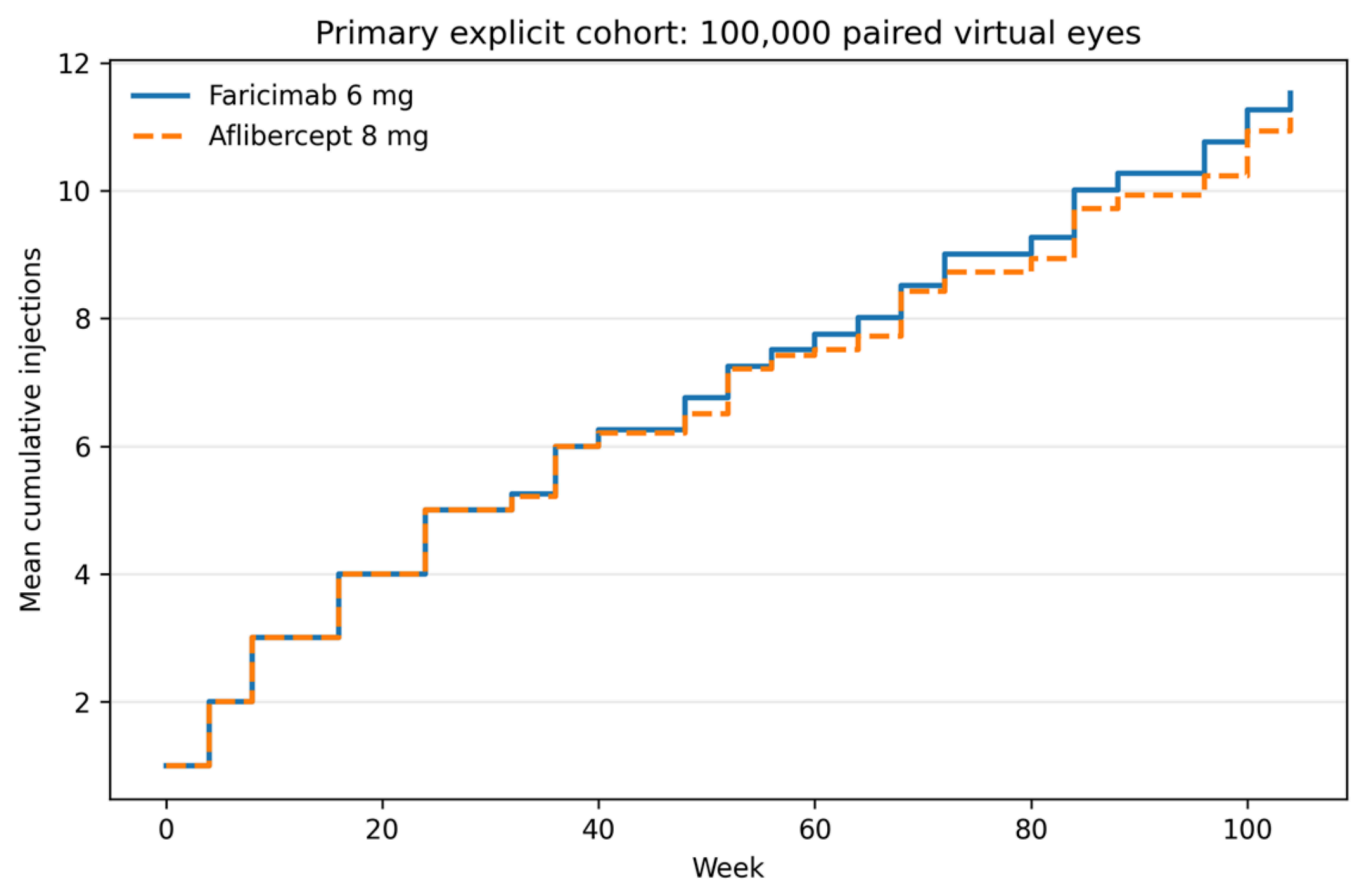
Mean cumulative injection trajectories through week 104 in the primary cohort of 100,000 paired virtual eyes. Each treatment-specific curve is determined by the simulated mixture of persistent Q8W, Q12W and Q16W capability classes under the identical protocol.

At week 104, expected injections were 11.53 with faricimab and 11.14 with aflibercept 8 mg. The fixed-weight difference was −0.39 injection (95% UI −0.68 to −0.11). Although 99.6% of draws favoured aflibercept 8 mg directionally, only 23.5% indicated at least 0.5 fewer injections, and virtually none indicated at least 1.0 fewer injections. Expected visits were 12.53 with faricimab and 12.14 with aflibercept 8 mg (Figure 2).

With a moderate cross-treatment capability-rank correlation of 0.5, aflibercept 8 mg required fewer injections in 31.15% of paired virtual eyes at week 104, both strategies required the same number in 55.41%, and faricimab required fewer injections in 13.44%. At week 52, the corresponding proportions were 14.8%, 74.78% and 10.43%.

### 2.4. Paired Virtual-Eye Microsimulation

The primary simulation generated all 100,000 requested paired virtual eyes. The maximum absolute deviation between the simulated and target marginal capability probabilities was 0.0023. The primary simulated week-104 contrast differed from its frozen analytic target by 0.0149 injection. The appraisal criteria are summarised in Supplementary Table S1.

At week 52, mean injections were 7.255 with faricimab and 7.212 with aflibercept 8 mg, giving a paired difference of −0.044 injection (Monte Carlo standard error 0.0016). Aflibercept 8 mg required fewer injections in 14.80% of virtual eyes, burden was equal in 74.78%, and faricimab required fewer injections in 10.43%. At week 104, the corresponding means were 11.522 and 11.144, with a difference of −0.378 injection (Monte Carlo standard error 0.0075). Aflibercept 8 mg required fewer injections in 31.15%, burden was equal in 55.41%, and faricimab required fewer injections in 13.44% (Table 2).

The nested analysis comprised 5000 outer draws and five million paired-eye evaluations. The exact outer-draw mean difference was −0.047 injection at week 52 (95% uncertainty interval −0.106 to 0.011) and −0.393 injection at week 104 (−0.682 to −0.107) (Table 2). The simulated week-104 contrast was −0.393036 compared with an exact outer-draw analytic contrast of −0.392671, giving a mean signed simulation error of −0.000365 injection. Across the five evidence specifications and both horizons, the maximum empirical spread in the mean contrast across correlation settings was 0.0196 injection; the corresponding analytic spread was zero because the correlation affects paired-eye outcomes but not marginal mean burden. All results were within the appraisal criteria summarised in Supplementary Table S1.

### 2.5. Evidence-Weight and Source-Pair Uncertainty

Under narrow source-weight uncertainty, the 104-week difference was −0.39 injection (95% UI −0.77 to −0.01). Under broad weight uncertainty, the mean remained −0.39 but the 95% UI crossed zero (−0.94 to 0.23). The corrected weight-centre audit passed all prespecified tolerances (Supplementary Figure S1).

**Figure 3:**
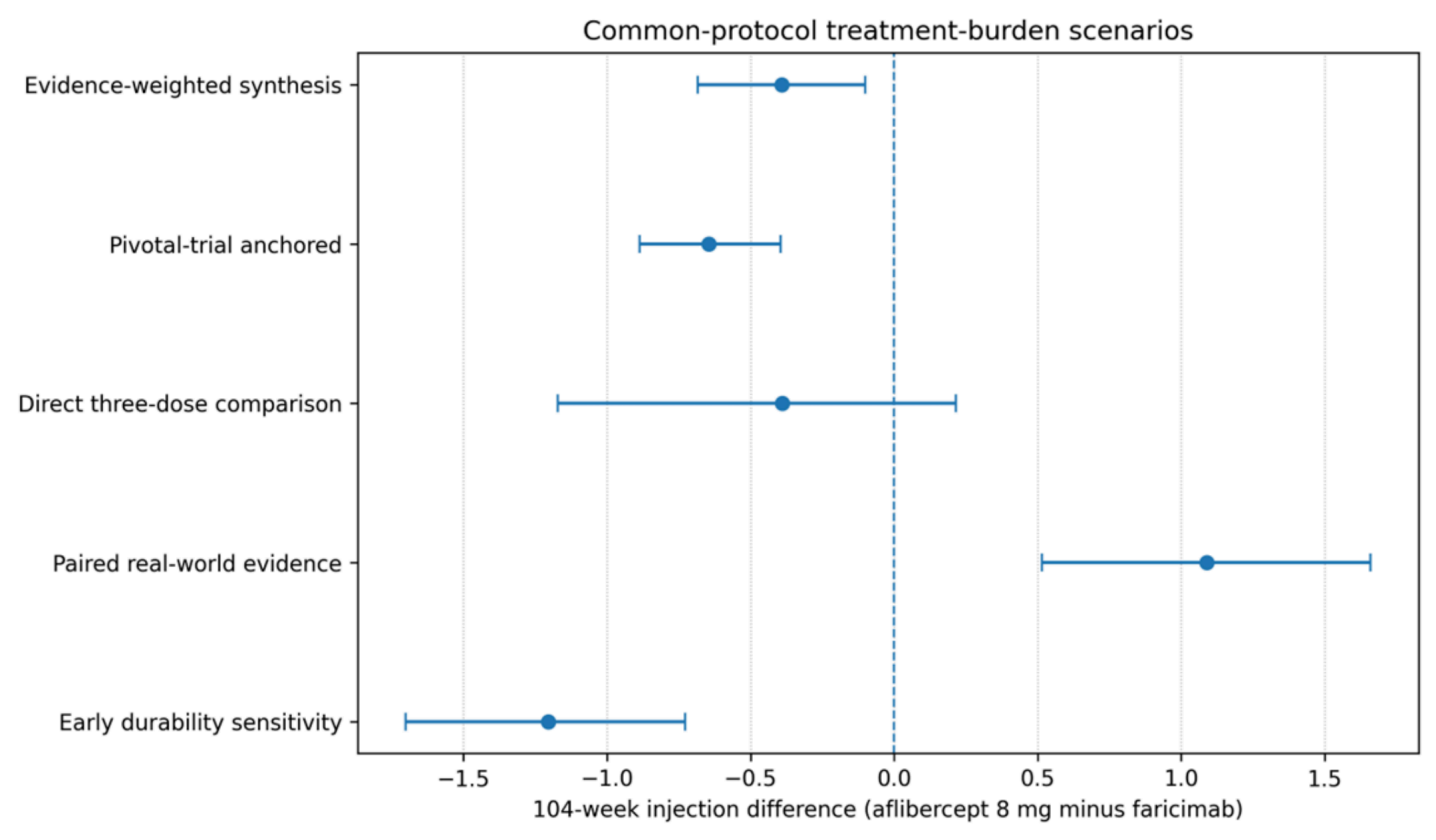
Prespecified 104-week common-protocol treatment-burden scenario envelope, displayed as a horizontal forest plot. Points show mean injection differences (aflibercept 8 mg minus faricimab) and lines show 95% uncertainty intervals. Negative values favour aflibercept 8 mg; positive values favour faricimab.

The estimated 104-week treatment-burden difference varied across the prespecified evidence scenarios. The pivotal-trial-anchored and early-durability scenarios estimated 0.65 and 1.20 fewer injections with aflibercept 8 mg, respectively, whereas the real-world pairing estimated 1.09 fewer injections with faricimab. The 95% uncertainty interval for the direct three-dose faricimab scenario included no difference. Thus, both the direction and magnitude of the modelled contrast varied according to the evidence specification (Table 3; Figure 3).

### 2.6. Leave-One-Source-Out Analysis

Omitting the three-dose faricimab durability source attenuated the difference to −0.10 injection and introduced substantial overlap with zero. Omitting the PULSAR-derived aflibercept 8 mg source changed the estimate to +0.71 injections (95% UI 0.19 to 1.24), favouring faricimab. In contrast, omitting the aflibercept 8 mg real-world source produced a difference of −0.78 injections (95% UI −1.07 to −0.49), favouring aflibercept 8 mg. The primary estimate was therefore influenced by the transportability assigned to the two aflibercept 8 mg sources (Supplementary Figure S2). The paired 104-week injection-difference distribution and the joint treatment-specific interval-capability distribution are shown in Supplementary Figures S3 and S4, respectively.

### 2.7. Exploratory Longitudinal Extension

#### 2.7.1. BCVA Trajectories and Evidence Appraisal

**Figure 4:**
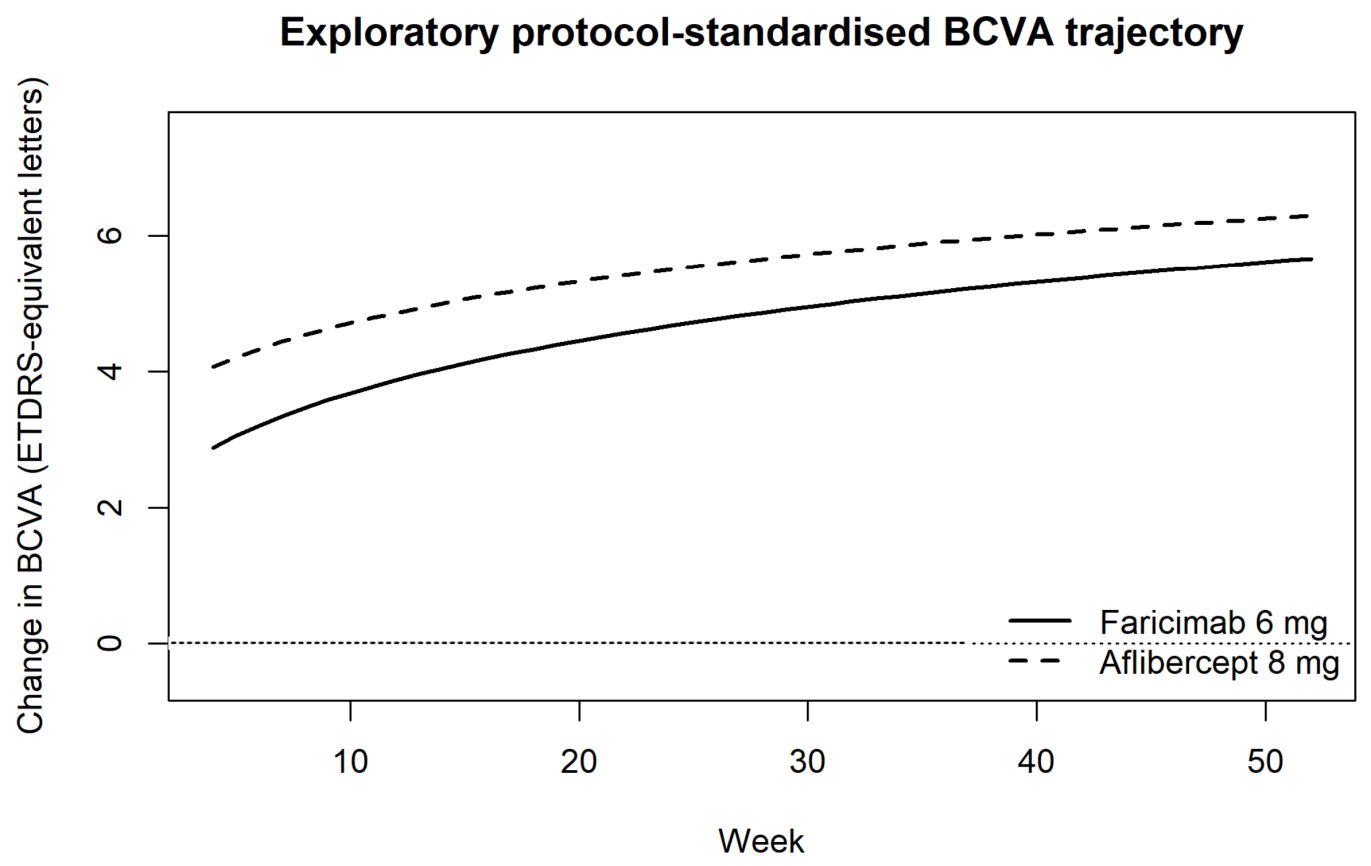
Model-based mean BCVA change from baseline over time for faricimab 6 mg and aflibercept 8 mg in treatment-naïve neovascular age-related macular degeneration, expressed in ETDRS or ETDRS-equivalent letters. The exploratory trajectories suggest broadly comparable visual outcomes between treatments.

The primary BCVA model included 29 aggregate observations from seven faricimab and three aflibercept 8 mg study families. At week 16, the projected gains were 4.19 ETDRS-equivalent letters with faricimab and 5.12 letters with aflibercept 8 mg; the aflibercept 8 mg-minus-faricimab difference was 0.93 letter (95% uncertainty interval −1.84 to 3.73). At week 52, the corresponding gains were 5.65 and 6.29 letters, with a difference of 0.64 letter (95% uncertainty interval −1.41 to 2.70; nominal *p* = 0.55) (Table 4; Figure 4). All alternative within-eye-correlation and three-dose analyses produced uncertainty intervals that included no difference, although the direction varied at the highest assumed correlation. RCT-only, real-world-only and native-ETDRS-only models could not be estimated under the prespecified family or observation thresholds, and omission of any aflibercept 8 mg family left fewer than three independent families. Detailed sensitivity and source-omission results are provided in Supplementary Table S2. The exploratory BCVA analysis did not indicate a material difference in mean visual outcomes, although the findings should be interpreted in the context of the available number of independent aflibercept 8 mg sources.

For external validation, the Turan three-dose loading gain was contained within the study-level predictive interval [12]. The FARWIDE treatment-naïve 12-month gain of 3.6 letters was marginally below the model predictive interval of 3.66 to 7.64 letters, a difference of 0.06 letter at the lower boundary [17].

#### 2.7.2. Retinal Thickness Trajectories

The strict CST/CRT model could not be estimated under the prespecified independent-family threshold. The expanded measurement-definition-adjusted sensitivity model included 41 observations from nine faricimab and three aflibercept 8 mg families and retained all 11 requested coefficients. At week 16, projected CST-equivalent changes were −160.9 micrometres with faricimab and −142.7 micrometres with aflibercept 8 mg; the difference was +18.2 micrometres (95% uncertainty interval −25.9 to 62.8). At week 52, changes were −171.8 and −147.2 micrometres, respectively, with a difference of +24.8 micrometres (95% uncertainty interval −20.7 to 69.2; nominal *p* = 0.29) (Table 4; Supplementary Figure S5). Positive contrasts indicate a larger reduction with faricimab.

Both treatments were associated with substantial projected reductions in retinal thickness. The magnitude and direction of the between-treatment contrast varied across covariance and loading-regimen assumptions, including a reversal in the three-dose-only analysis. The strict CST/CRT-only model could not be estimated under the prespecified evidence threshold. Detailed sensitivity results are provided in Supplementary Table S2; the anatomical contrast should therefore be regarded as supportive and hypothesis-generating.

#### 2.7.3. Retinal Fluid Outcomes

IRF-absence and SRF-absence findings were presented descriptively because each analysis contained one eligible aflibercept 8 mg family. Complete retinal dryness included 13 aggregate observations and three independent families per treatment. The projected probabilities were 45.9% (95% uncertainty interval 39.1–52.8%) with faricimab and 64.1% (60.6–67.5%) with aflibercept 8 mg at week 16, and 57.0% (48.6–65.1%) and 69.0% (65.0–72.7%), respectively, at week 52 (Table 4; Supplementary Figure S6). These estimates provided supportive exploratory information and complemented the visual and retinal thickness findings. They should be interpreted in the context of the available number of independent study families and variation in dryness definitions, rather than as evidence of anatomical superiority.

## 3. Discussion

In this protocol-standardised evidence synthesis and paired virtual-eye microsimulation, faricimab and aflibercept 8 mg produced essentially indistinguishable treatment burden at one year. The 100,000-paired-eye cohort estimated only 0.04 fewer injections with aflibercept 8 mg at week 52. At week 104, the paired cohort estimated 0.38 fewer injections and the nested parameter analysis estimated 0.39 fewer injections. These results had low Monte Carlo error, but their clinical magnitude was small and their direction remained sensitive to the evidence sources considered transportable.

The one-year result follows from use of the same treatment schedule. Under the common protocol, both Q12W- and Q16W-capable eyes receive seven injections by approximately one year; only the Q8W subgroup materially affects the first-year mean. At two years, Q8W-, Q12W- and Q16W-capable eyes receive 15, 11 and 10 injections, respectively, so persistent interval heterogeneity becomes more influential. The expected two-year contrast can be decomposed into four times the between-treatment difference in Q8W capability minus the difference in Q16W capability. This explains why modest uncertainty about the frequent-treatment subgroup can outweigh uncertainty about the distinction between Q12W and Q16W.

The common protocol is not necessarily neutral with respect to the regimens used in the pivotal trials. Its three-dose loading phase aligns with PULSAR, whereas TENAYA and LUCERNE used four initial Q4W faricimab doses; the effect of this difference on the comparative estimates is uncertain. Conversely, the Q16W ceiling prevents the model from capturing the Q20W and Q24W intervals available to eligible aflibercept 8 mg-treated eyes during the second year of PULSAR and may therefore understate its longer-term durability. The mandatory week-16 injection under the common protocol also limits the opportunity for early separation in injection burden. These design features could affect the two treatments differently, but their net effect is unknown. The findings therefore address a protocol-specific counterfactual question and should not be interpreted as a protocol-independent comparison of treatment durability.

The present estimate is substantially smaller than the approximately 1.47-injection two-year advantage for aflibercept 8 mg reported by a recent Bayesian network meta-analysis [10]. That network meta-analysis retained the original trial schedules, including four faricimab loading injections and the separate TENAYA/LUCERNE and PULSAR maintenance rules. An economic analysis based on the original trial injection counts similarly projected lower longer-term treatment burden and costs with aflibercept 8 mg [11]. The attenuation observed here suggests that an important component of the published cross-trial burden difference is attributable to regimen architecture rather than unequivocal molecular durability.

The exploratory longitudinal extension provides supportive functional context but does not convert the study into a comparative efficacy analysis. The projected week-52 BCVA difference was only 0.64 letter and every correlation or loading-regimen uncertainty interval included no difference. The result was informed by three aflibercept 8 mg study families, and the native-ETDRS-only analysis could not be estimated under the prespecified threshold. The appropriate interpretation is therefore that the aggregate evidence did not reveal a material visual difference, not that visual equivalence or non-inferiority was demonstrated.

The anatomical projections should be viewed as hypothesis-generating. The strict CST/CRT model could not be estimated under the prespecified family threshold, and the expanded CST-equivalent model combined related but non-identical measurement definitions. Its nominal faricimab-favouring direction differed in the three-dose-only analysis. Complete retinal dryness was numerically higher with aflibercept 8 mg in the fitted marginal projections, but the estimates were based on three families per treatment and combined study-specific dryness definitions; source-omission analysis was therefore not feasible. These secondary results do not establish anatomical superiority.

The latent interval-capability classes reflect the limitations of the aggregate evidence and should not be interpreted as fixed biological states or as an assumption that treatment intervals do not fluctuate clinically. A dynamic recurrence or multistate formulation was considered, but the published aggregate data did not provide the eye-level transition probabilities or longitudinal relationships among retinal fluid, BCVA and retreatment decisions needed to parameterise such a model without strong unverifiable assumptions. The Q8W, Q12W and Q16W classes therefore represent each simulated eye’s predominant interval requirement over the model horizon. Individual-patient longitudinal data would be needed to estimate extension and shortening transitions directly.

A strength of the study is the separation of protocol effects, evidence-source assumptions, parameter uncertainty and Monte Carlo error. Structured model-appraisal checks, documented key inputs and source-influence analyses allow the sensitivity of the results to the principal modelling assumptions and evidence sources to be assessed.

The study has several limitations. First, it used aggregate rather than individual-patient data, so correlations among baseline BCVA, fluid phenotype, treatment response, interval extension and longer-term burden could not be estimated directly. Second, combining randomised and observational evidence required judgements about the transportability of study populations, healthcare pathways, imaging thresholds and retreatment decisions to the target framework. The relevance weights captured these judgements in a structured way but could not remove residual between-study differences, and the broad-weight, source-pair and source-omission analyses showed that the two-year estimate was sensitive to source selection. Third, the longitudinal analysis relied on approximate conversion from logMAR to ETDRS letters, reconstruction of change-score variances and repeated-measure covariance under assumed within-eye correlations, and study-family grouping to account for sequential or potentially overlapping reports.

Further limitations relate to the exploratory efficacy and anatomical analyses and to outcomes that were not modelled. Only three independent aflibercept 8 mg study families contributed to the one-year BCVA model, preventing a complete leave-one-family-out assessment; consequently, the visual projections cannot establish equivalence or non-inferiority. The strict CST/CRT and native-ETDRS-only BCVA models did not meet the minimum evidence thresholds. The expanded anatomical analysis combined related but non-identical retinal thickness definitions, although measurement type was included as a moderator, while variation in definitions of dryness and central-fluid control limited the comparability of fluid outcomes. Comparative efficacy at week 104 remained descriptive because longer-term aflibercept 8 mg evidence was dominated by the PULSAR study family. Safety and economic outcomes, including treatment-emergent adverse events, intraocular inflammation, systemic safety, drug acquisition costs and service-specific monitoring costs, were not assessed.

Clinically, the results do not support choosing between faricimab and aflibercept 8 mg solely on the expectation of a large injection-burden difference when both are assessed under the same protocol. At one year, the model predicts virtually identical burden. At two years, aflibercept 8 mg has a modest advantage under trial-dominant weights, but the conclusion changes when real-world evidence is given greater weight. Treatment selection should continue to incorporate individual anatomical response, interval achieved after loading, safety, local availability, cost and patient preference rather than assuming a universal durability hierarchy.

Prospective head-to-head studies using identical loading and treat-and-extend criteria remain the most reliable way to resolve the comparison. Short of such a trial, individual-patient-data models could combine serial OCT and OCT angiography biomarkers, baseline phenotype, treatment exposure and clinical covariates within multistate or machine-learning frameworks. Recent studies in diabetic retinal disease have highlighted the potential of artificial intelligence and longitudinal imaging analyses for predicting anti-VEGF response and characterising structural changes over time [18,19]. These studies provide broader methodological context rather than direct evidence for nAMD. Applying similar approaches to nAMD could link early fluid response with later interval attainment and identify clinically meaningful responder phenotypes, but external validation across treatment pathways and healthcare systems would be essential. The direct three-dose loading comparison provides an important early efficacy bridge [15], although longer follow-up under aligned retreatment rules is still required.

## 4. Materials and Methods

### 4.1. Study Design and Analytic Framework

This was a probabilistic aggregate-data evidence-synthesis and paired virtual-eye microsimulation study under a protocol-standardised treatment schedule. Published trial and real-world outcomes were used to construct treatment-specific distributions of persistent interval capability. The common protocol was applied both analytically and to individual virtual eyes. The final simulation comprised a 100,000-paired-eye primary cohort, a 1.5-million-pair scenario and correlation grid, and a nested probabilistic analysis containing five million paired-eye evaluations. Each virtual eye was assessed counterfactually under both treatments; no simulated eye was treated as an independently observed clinical participant.

The aggregate reports were not sufficiently granular to support reliable estimation of a dynamic state-transition model, because eye-level interval transitions, recurrent disease activity and the joint longitudinal relationships among retinal fluid, BCVA and retreatment decisions were not consistently reported. An alternative recurrence-time model was also assessed but did not adequately reproduce the held-out interval-transition patterns. The final analysis therefore used latent Q8W, Q12W and Q16W capability classes as a parsimonious summary of each eye’s predominant treatment requirement over the model horizon. These classes are modelling constructs rather than fixed biological states and do not imply that treatment intervals remain constant in clinical practice.

### 4.2. Evidence Identification, Eligibility and Hierarchy

Evidence was assembled through a targeted, model-component-specific search rather than a formal systematic review. PubMed, publisher websites, pivotal trial reports and their supplementary appendices were searched through 16 July 2026, supplemented by backward and forward citation review. Search concepts combined the treatment names and doses with neovascular age-related macular degeneration, treatment-naïve disease, loading regimen, treat-and-extend, durability, injection interval, BCVA, retinal thickness, intraretinal fluid and subretinal fluid. Eligible evidence comprised randomised trials or treatment-naïve observational cohorts reporting extractable aggregate data on loading response, injection frequency, achieved intervals, BCVA, retinal thickness or retinal fluid outcomes. Mixed or previously treated populations were used only when treatment-naïve nAMD results were available separately. Sequential reports and probable overlapping cohorts were assigned a common study-family identifier, and abstract-only evidence was restricted to external plausibility assessment. The search was designed to identify evidence for prespecified model components and was not conducted or reported as a PRISMA-compliant systematic review.

The evidence hierarchy comprised: (1) pivotal randomised trials and their complete supplementary appendices; (2) three-dose faricimab loading and durability cohorts; (3) direct three-versus-four-dose faricimab evidence; and (4) treatment-naïve real-world cohorts for external validation and pragmatic sensitivity analyses. TENAYA/LUCERNE and PULSAR informed original-regimen assessment and interval distributions [6,7,8,9]. Faricimab three-dose loading response and durability were informed by Turan et al., Han et al. and Doi et al. [12,13,14]. Australian faricimab and Swiss aflibercept 8 mg cohorts informed pragmatic capability scenarios [20,21]. The evidence sources and their assigned roles are summarised in Table 5.

Source relevance weights were defined and fixed before the final comparative simulations. They reflected the applicability of each evidence source to the treatment-naïve target population and common protocol, rather than representing posterior probabilities or formal risk-of-bias scores. Weighting considered compatibility with the loading and extension framework, directness of the interval or durability endpoint, study design, follow-up duration, completeness of interval reporting and potential cohort overlap. Because these transportability judgements are not uniquely determined from aggregate data, their influence was examined using narrow and broad weight distributions, alternative source-pair scenarios and leave-one-source-out analyses. Detailed statistical models and exploratory longitudinal sensitivity analyses are provided in Supplementary Tables S1 and S2, respectively; source-specific weights and their rationale are provided in Supplementary Table S3. The corresponding observed simulation implementation and Monte Carlo appraisal results are provided in Supplementary Table S4.

BCVA and anatomical variables were retained as supporting evidence. The pooled faricimab three-dose loading input was a mean gain of 5.11 ETDRS letters, a mean central retinal thickness reduction of 156.5 micrometres, and a probability of complete central fluid control of 79.9%. PULSAR provided week-16 central fluid control of 62% and 65% in the Q12W and Q16W groups, respectively, with week-48 BCVA gains of 6.7 and 6.2 letters and mean retinal thickness reductions of 142 and 147 micrometres [7]. A direct three-dose loading comparison reported no significant between-treatment differences in BCVA improvement, retinal thickness reduction or complete retinal fluid resolution [15]. These parameters were used to assess clinical plausibility and external model performance; they were not used to generate a comparative common-protocol claim of visual or anatomical superiority.

### 4.3. Common Treatment Protocol

Both strategies received intravitreal injections at weeks 0, 4 and 8. A monitoring assessment was included at week 12, followed by a mandatory maintenance injection at week 16. Thereafter, all eyes began at Q8W. Eyes capable of longer control extended in four-week steps to Q12W and then Q16W; the maximum modelled interval was Q16W. The interval pathway was identical for both molecules.

**Figure 5:**
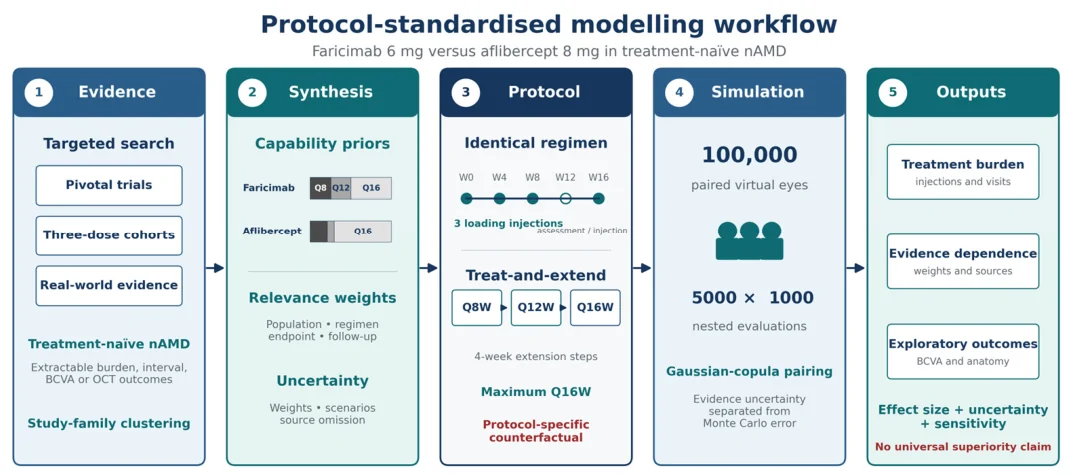
Protocol-standardised modelling workflow. Targeted evidence from pivotal trials, three-dose cohorts and treatment-naïve real-world studies informed treatment-specific Q8W/Q12W/Q16W capability distributions. The same loading and extension protocol was applied to 100,000 paired virtual eyes, with nested uncertainty and source-sensitivity analyses. The comparison is specific to the modelled protocol and is not intended as a protocol-independent ranking of intrinsic molecular durability.

Under this schedule, Q8W-, Q12W- and Q16W-capable eyes received 8, 7 and 7 injections, respectively, through week 52. Through week 104, the corresponding totals were 15, 11 and 10 injections. The common protocol therefore provides relatively little separation between Q12W- and Q16W-capable eyes during the first year but greater differentiation during the second year. A schematic overview of the evidence inputs, latent capability model, common protocol and analysis outputs is provided in Figure 5.

### 4.4. Statistical Analysis

The primary estimand was the difference between aflibercept 8 mg and faricimab 6 mg in cumulative injections through weeks 52 and 104 under the common protocol. Interval-capability distributions were informed by treatment-naïve evidence sources in which mean visual outcomes were maintained under the original study regimens. Because the primary model did not simulate longitudinal BCVA, the treatment-burden estimates do not constitute a test of visual equivalence or non-inferiority under the common protocol. Secondary outcomes included cumulative monitoring visits, Q8W, Q12W and Q16W capability probabilities, and the paired probabilities that aflibercept 8 mg required fewer, the same number of, or more injections than faricimab. Unless otherwise stated, treatment contrasts were defined as aflibercept 8 mg minus faricimab; negative values indicate fewer injections with aflibercept 8 mg.

Published randomised and real-world evidence was used to derive treatment-specific distributions of sustained Q8W, Q12W and Q16W capability. Candidate recurrence-time models were first calibrated against reported loading, interval and injection outcomes and assessed against held-out long-term targets. A homogeneous recurrence model did not meet those checks and was therefore not carried forward to the comparative analysis. The final treatment-burden model used persistent interval-capability classes. Both treatments were then assigned the same schedule: injections at weeks 0, 4 and 8, assessment at week 12, a mandatory injection at week 16, and subsequent treat-and-extend dosing from Q8W to Q16W in four-week increments. Conditional on capability class, injection and visit schedules were fixed. The principal interval-capability and common-protocol burden outputs are summarised in Table 1.

The primary microsimulation comprised 100,000 counterfactually paired virtual eyes. Each eye received a potential interval-capability class under both treatments, allowing within-eye comparison of the two common-protocol strategies. Treatment-specific marginal capability distributions were coupled with a Gaussian copula using a rank correlation of 0.50 in the primary analysis; correlations of 0 and 1.00 were examined separately. The assumed correlation affects paired-eye probabilities but not treatment-specific mean burden. The virtual cohort represented treatment-burden states rather than complete clinical phenotypes, and the primary model did not generate individual longitudinal BCVA or OCT trajectories.

Capability uncertainty was carried through 30,000 source-level draws. The primary comparison used prespecified relevance weights, while narrow and broad Dirichlet distributions assessed uncertainty in those weights. Five evidence specifications and leave-one-source-out analyses examined dependence on trial, three-dose and real-world inputs. A nested probabilistic analysis used 5000 outer parameter draws and 1000 paired eyes per draw. Class-probability-weighted burden was calculated analytically within each outer draw, while the inner simulations estimated paired-eye outcome probabilities and Monte Carlo error. Results are reported as means with 95% uncertainty intervals; Monte Carlo standard errors are shown separately where informative. The prespecified scenario analyses and the overall interpretive framework are summarised in Table 3 and Table 6, respectively; corresponding graphical assessments are provided in Supplementary Figures S1 and S2.

An exploratory longitudinal extension modelled aggregate outcomes for BCVA, retinal thickness and fluid at weeks 16 and 52. Multilevel meta-regression accounted for repeated observations from the same study family and included treatment, follow-up time, baseline outcome and loading regimen. BCVA was analysed as ETDRS or ETDRS-equivalent change. The primary retinal thickness specification was restricted to CST or CRT; broader thickness definitions were permitted only in a measurement-adjusted sensitivity analysis. IRF absence, SRF absence and complete retinal dryness were analysed as separate binomial outcomes. Nominal two-sided model-based *p* values were calculated for the prespecified week-52 BCVA and CST-equivalent retinal thickness contrasts using the t reference distribution from the fitted meta-regression. These *p* values were secondary, were not adjusted for multiplicity and were interpreted together with effect sizes, uncertainty intervals and sensitivity analyses. Week-104 visual and anatomical values were retained as descriptive anchors because long-term aflibercept 8 mg evidence was dominated by the PULSAR programme.

Interpretation was based on effect magnitude, 95% uncertainty intervals, consistency across evidence specifications, source-omission analyses and prespecified model checks. No *p* value was calculated from the number of virtual eyes because the simulated cohort does not add clinical information; *p* values were confined to the two selected week-52 exploratory aggregate-data meta-regression contrasts. The exploratory longitudinal findings were described according to whether they met the prespecified checks, provided supportive evidence within the available evidence base, required broader outcome definitions, varied across sensitivity analyses or were best presented descriptively. A treatment advantage was not claimed when credible analyses changed the direction of the contrast.

Analyses were performed in R version 4.6.1. The longitudinal extension used metafor for multilevel meta-regression, clubSandwich for CR2 small-sample-adjusted covariance analyses, mvtnorm for coefficient simulation and Matrix for covariance repair. Random-number seeds were fixed. Full equations, transformations, source distributions, parameter settings, simulation checks, model-appraisal rules and software details are provided in Supplementary Appendix S1–S10 and Supplementary Tables S1–S3.

## 5. Conclusions

When faricimab 6 mg and aflibercept 8 mg were modelled with identical three-injection loading and Q8W-to-Q16W extension protocols, the 100,000-paired-eye simulation predicted essentially similar treatment burden at 52 weeks. At 104 weeks, the fixed evidence synthesis and nested probabilistic analysis modestly favoured aflibercept 8 mg by approximately 0.4 injection, but the absolute difference was small, broad source-weight uncertainty included no difference, and credible real-world and source-omission analyses changed the direction of the contrast. The exploratory longitudinal projections were based on only three independent aflibercept 8 mg study families at one year and should be interpreted as hypothesis-generating. They did not establish visual equivalence, non-inferiority or anatomical superiority. Overall, the available evidence did not establish protocol-independent treatment-burden, visual or anatomical superiority of either treatment.

## Figures and Tables

**Table 1:** Primary interval-capability distributions and common-protocol treatment burden.

Outcome	Faricimab	Aflibercept 8 mg	Difference/Contrast
Q8W capability	25.7% (95% UI 21.3–30.4%)	21.1% (17.4–25.1%)	−4.6 percentage points for A8
Q12W capability	24.5% (20.6–28.8%)	8.6% (6.0–11.6%)	−15.9 percentage points for A8
Q16W capability	49.8% (44.6–54.9%)	70.3% (65.8–74.6%)	+20.6 percentage points for A8
Injections through week 52	7.26	7.21	A8-FAR −0.05 (95% UI −0.11 to 0.01)
Visits through week 52	8.26	8.21	A8-FAR −0.05
Injections through week 104	11.53	11.14	A8-FAR −0.39 (95% UI −0.68 to −0.11)
Visits through week 104	12.53	12.14	A8-FAR −0.39

**Table 2:** Paired virtual-eye and nested probabilistic microsimulation results.

Analysis	Horizon	Faricimab	Aflibercept 8 mg	Difference or Probability	Interpretation
Primary paired virtual-eye cohort	52 weeks	7.255 injections	7.212 injections	A8-FAR −0.044; MCSE 0.0016	Essentially equal burden
Primary paired virtual-eye cohort	104 weeks	11.522 injections	11.144 injections	A8-FAR −0.378; MCSE 0.0075	Small mean reduction with A8
Paired eye-level outcomes	104 weeks	Fewer in 13.44%	Fewer in 31.15%	Equal in 55.41%	Most virtual eyes had equal burden
Nested parameter analysis	52 weeks	7.258 expected	7.211 expected	−0.047 (95% UI −0.106 to 0.011)	Includes no difference
Nested parameter analysis	104 weeks	11.533 expected	11.140 expected	−0.393 (95% UI −0.682 to −0.107)	Fixed synthesis modestly favours A8

**Table 3:** Prespecified 104-week scenario and sensitivity analyses.

Analysis	Difference (A8-FAR)	95% UI	Interpretation
Fixed evidence-weighted synthesis	−0.39	−0.68 to −0.11	Modestly fewer injections with A8 under frozen weights
Narrow source-weight uncertainty	−0.39	−0.77 to −0.01	Direction retained but attenuated
Broad source-weight uncertainty	−0.39	−0.94 to 0.23	Includes no difference
RCT-anchored scenario	−0.65	−0.89 to −0.40	Fewer injections with A8
Direct three-dose FAR scenario	−0.39	−1.17 to 0.22	Uncertain
Real-world paired scenario	+1.09	0.52 to 1.66	Fewer injections with faricimab
Early FAR durability scenario	−1.20	−1.70 to −0.73	Fewer injections with A8
Omit PULSAR-derived A8 source	+0.71	0.19 to 1.24	Direction reverses; faricimab fewer
Omit A8 real-world source	−0.78	−1.07 to −0.49	A8 fewer

**Table 4:** Exploratory longitudinal projections of visual outcomes, retinal thickness and complete retinal dryness.

Outcome/Horizon	Faricimab 6 mg	Aflibercept 8 mg	A8 Minus Faricimab	Interpretation
BCVA, week 16	+4.19 letters (95% UI 1.82 to 6.57)	+5.12 letters (3.88 to 6.37)	+0.93 letter (−1.84 to 3.73)	Supportive exploratory finding; small, uncertain contrast
BCVA, week 52	+5.65 letters (3.81 to 7.50)	+6.29 letters (5.36 to 7.21)	+0.64 letter (−1.41 to 2.70); *p* = 0.55	Supportive exploratory finding; no material visual difference demonstrated
CST-equivalent change, week 16	−160.9 micrometres (−192.5 to −129.5)	−142.7 micrometres (−191.2 to −94.4)	+18.2 micrometres (−25.9 to 62.8)	Hypothesis-generating anatomical sensitivity analysis
CST-equivalent change, week 52	−171.8 micrometres (−202.6 to −141.1)	−147.2 micrometres (−195.9 to −98.4)	+24.8 micrometres (−20.7 to 69.2); *p* = 0.29	Hypothesis-generating; direction varied in the three-dose-only analysis
Complete retinal dryness, week 16	45.9% (39.1–52.8%)	64.1% (60.6–67.5%)	+18.2 percentage points (descriptive)	Supportive exploratory finding; three families per treatment
Complete retinal dryness, week 52	57.0% (48.6–65.1%)	69.0% (65.0–72.7%)	+11.9 percentage points (descriptive)	Supportive exploratory finding; interpreted in the context of the available evidence base

**Table 5:** Visual, anatomical and durability evidence used for model construction and assessment.

Evidence Source	Regimen/Population	Visual Outcome	Anatomical Outcome	Burden/Durability Outcome	Model Role
TENAYA/LUCERNE [6,8]	Faricimab 6 mg; four monthly loading doses; original protocol	Week-48 BCVA +5.8/+6.6 letters; sustained through week 112	Week-48 CST −136.8/−129.4 micrometres	Week-48 Q8W/Q12W/Q16W approximately 21%/33%/45%	Original-regimen assessment; pivotal interval scenario
PULSAR [7,9]	Aflibercept 8 mg; three monthly loading doses; Q12W or Q16W with modification	Week-48 BCVA +6.7/+6.2 letters; week-96 +5.6/+5.5	Week-16 fluid-free 62%/65%; week-48 CRT −142/−147 micrometres	Mean injections 6.1/5.2 to week 48 and 9.7/8.2 to week 96	Primary aflibercept 8 mg capability source
Three-dose faricimab cohorts [12,13]	Three monthly injections in treatment-naïve nAMD	Pooled loading input +5.11 ETDRS letters	Pooled CST reduction −156.5 micrometres; complete fluid control approximately 79.9%	Schedule-adjusted durability after third injection	Loading plausibility and early durability
Direct 3-dose comparison [15]	Aflibercept 8 mg n = 51; faricimab n = 49	No significant difference in BCVA improvement	No significant difference in CRT reduction; complete fluid resolution 80.4% vs. 87.8%	Loading phase only	External functional/anatomical guardrail
Doi et al. [14]	Three versus four faricimab loading injections	Comparable one-year functional outcome	Comparable one-year CMT outcome	Three-dose Q8W/Q12W/Q16W 4/25, 6/25, 15/25	Direct three-dose sensitivity
Australian FAR and Swiss A8 RWE [20,21]	Treatment-naïve routine clinical practice	Visual gains maintained at 12 months	Anatomical improvement and fluid control	Real-world interval distributions	Pragmatic source-pair sensitivity
Longitudinal faricimab RWE [17,22,23,24,25,26,27]	Three- or four-dose loading; treatment-naïve cohorts; repeated outcomes to 12–24 months	Repeated BCVA/logMAR or ETDRS outcomes	CST/CRT/CMT/foveal thickness and fluid outcomes where reported	Injection exposure and achieved intervals	Exploratory longitudinal model, external validation and sensitivity analyses
Longitudinal aflibercept 8 mg RWE [21,28,29,30,31]	Treatment-naïve early and one-year cohorts	Repeated BCVA outcomes	CST/CRT/CMT, IRF/SRF and dryness outcomes	Loading and one-year injections/intervals	Exploratory longitudinal model and measurement-definition sensitivity

**Table 6:** Overview of statistical analysis and interpretive role.

Analysis Component	Main Method	Purpose	Main Restriction
Evidence synthesis	Treatment-specific Q8W/Q12W/Q16W capability distributions derived from trial and real-world evidence.	Represent the interval mix expected under the target population and protocol.	Source weights reflect relevance rather than formal probabilities that a study is correct.
Primary estimand	Difference in cumulative injections and visits through weeks 52 and 104 under the same loading and maintenance schedule, using capability distributions derived from sources with maintained mean visual outcomes.	Separate treatment effects from protocol differences.	No new eye-level BCVA threshold was imposed; visual control was appraised at the source-study population level.
Paired virtual-eye simulation	100,000 counterfactually paired eyes coupled by a Gaussian copula.	Estimate mean burden and paired probabilities of fewer, equal or greater injections.	Virtual-eye number controls Monte Carlo precision and is not a clinical sample size.
Parameter uncertainty	Source-level capability draws, uncertain evidence weights and a nested 5000 × 1000 paired-eye analysis.	Propagate uncertainty from the aggregate evidence base.	Uncertainty intervals are not conventional confidence intervals from a head-to-head trial.
Scenario and influence analyses	Trial-anchored, three-dose, real-world and early-durability specifications, plus leave-one-source-out analyses.	Assess dependence on evidence selection and transportability.	A treatment advantage is not claimed when credible specifications change direction.
Exploratory longitudinal extension	Multilevel aggregate-data models for BCVA, retinal thickness and separate fluid endpoints.	Provide secondary protocol-standardised visual and anatomical projections.	Results are hypothesis-generating; week-104 comparisons are descriptive.
Evidence appraisal and reporting	Convergence, family-count, sensitivity, source-omission and external-prediction checks.	Describe whether each result met the prespecified exploratory checks, provided supportive evidence within the available evidence base, required broader outcome definitions, varied across sensitivity analyses or was best presented descriptively.	No *p* values are derived from the number of simulated eyes; nominal *p* values are confined to exploratory continuous meta-regression contrasts.

## Data Availability

All data used in this study were derived from published aggregate sources cited in the article. No new patient-level data were generated. Derived datasets, analysis code, simulation scripts and model outputs are available from the corresponding author on reasonable request.

## Supplementary Materials

The following supporting information can be downloaded at: https://www.mdpi.com/article/10.3390/ph19081316/s1, Supplementary Appendix S1. Evidence extraction, transformations and study-family structure; Supplementary Appendix S2. Supporting loading-response synthesis; Supplementary Appendix S3. Preliminary recurrence-time calibration and model refinement; Supplementary Appendix S4. Latent interval-capability model and evidence synthesis; Supplementary Appendix S5. Common-protocol estimands and treatment-burden calculations; Supplementary Appendix S6. Evidence-source uncertainty and influence analyses; Supplementary Appendix S7. Paired virtual-eye microsimulation and Monte Carlo assessment; Supplementary Appendix S8. Exploratory longitudinal aggregate-data models; Supplementary Appendix S9. Evidence-appraisal rules and inferential conventions; Supplementary Appendix S10. Software and computational implementation; Supplementary Table S1. Detailed statistical models, simulations and model appraisal rules; Supplementary Table S2. Exploratory longitudinal contrasts and principal sensitivity analyses; Supplementary Table S3. Prespecified evidence-relevance weights and transportability rationale; Supplementary Table S4. Simulation implementation and Monte Carlo appraisal results; Supplementary Figure S1. Evidence-weight uncertainty around the fixed 104-week source weights; Supplementary Figure S2. Leave-one-source-out influence analysis at week 104; Supplementary Figure S3. Distribution of the paired 104-week injection difference in the primary virtual-eye cohort, defined as aflibercept 8 mg minus faricimab; Supplementary Figure S4. Joint treatment-specific interval-capability distribution in the primary cohort at a Gaussian-copula rank correlation of 0.50; Supplementary Figure S5. Model-based CST-equivalent retinal thickness trajectories from the expanded measurement-definition sensitivity analysis; Supplementary Figure S6. Exploratory complete retinal dryness probabilities.
