# Supplementary material for: Faricimab 6 mg Versus Aflibercept 8 mg in Treatment-Naïve Neovascular Age-Related Macular Degeneration: A Protocol-Standardised In Silico Study
Source: Pharmaceuticals (Basel). 2026 Aug 20;19(8):1316. doi: 10.3390/ph19081316 (PMC13516086; doi:10.3390/ph19081316)

Supplementary Statistical Appendix

Faricimab 6 mg versus Aflibercept 8 mg in Treatment-Naïve Neovascular Age-Related Macular Degeneration: A Protocol-Standardised In Silico Study

Supplementary Appendix S1. Evidence extraction, transformations and study-family structure

The clinical evidence base was assembled through a targeted, model-component-specific search rather than a formal systematic review. PubMed, publisher websites, pivotal trial reports and supplementary appendices were searched through 16 July 2026, with backward and forward citation review. Search concepts combined the drug names and doses with nAMD, treatment-naïve disease, loading, treat-and-extend, durability, interval, BCVA, retinal thickness, IRF and SRF. Eligible sources were randomised trials or treatment-naïve observational cohorts with extractable aggregate data relevant to loading response, interval capability, treatment burden or longitudinal visual/anatomical outcomes. Mixed populations were used only when treatment-naïve nAMD results were separable. The search was intended to populate prespecified model components and was not a PRISMA-compliant systematic review.

Numerical outcomes were transcribed from the main articles and supplementary files at every usable time point. Continuous variables were recorded as means with standard deviations, standard errors or 95% confidence intervals. When only a 95% confidence interval was available, the standard error was calculated as (upper limit - lower limit)/(2 x 1.96). Binary outcomes were recorded as events and denominators. Treatment-naïve groups were kept separate from previously treated groups. No unreported outcome value was imputed. Rows with a mean but no defensible variance estimate were retained as descriptive or external model-assessment anchors and were excluded from inverse-variance models.

Reports arising from the same cohort, sequential follow-up of the same participants or a probable overlapping clinical network were assigned a common study-family identifier. Such reports could contribute distinct time points or outcomes, but they were not counted as independent study families. The faricimab and aflibercept 8 mg Matsumoto loading and one-year reports, the faricimab one- and two-year reports, and the Swiss treatment-specific series were handled in this manner. Duplicate baseline or follow-up observations from continuation reports were excluded from quantitative fitting.

For supportive loading-response analyses, logMAR change was converted to approximate ETDRS letter change as -50 x change in logMAR. A missing change-score standard deviation was reconstructed as $\text{sqrt}(SD_{baseline}^2 + SD_{follow\text{-}up}^2 - 2r \times SD_{baseline} \times SD_{follow\text{-}up})$, with r=0.50. For the exploratory longitudinal BCVA model, baseline logMAR was converted to an ETDRS-equivalent value as 85 - 50 x logMAR, and the primary within-eye correlation for reconstructed change scores was r=0.60, with r=0.30 and r=0.90 examined separately. Positive BCVA values denote letter gain; negative retinal-thickness values denote thinning from baseline.

Supplementary Appendix S2. Supporting loading-response synthesis

Three-dose faricimab BCVA and retinal-thickness inputs were combined using relevance-weighted inverse-total-variance means. For source i, w_i was proportional to $R_i/(SE_i^2 + h^2)$, where R_i was the prespecified relevance multiplier and h was a heterogeneity floor of 1.5 ETDRS letters for BCVA and 15 micrometres for retinal thickness. Weights were normalised to sum to one. The non-randomised, centre-confounded three-versus-four dose comparison received a lower relevance multiplier. The pooled complete-fluid-control probability was obtained by Beta-binomial updating with a Beta(1,1) prior; the smaller corroborative cohort entered as a fractional-likelihood contribution. These quantities served only as loading-phase plausibility checks and did not determine the final treatment-burden contrast.

Supplementary Appendix S3. Preliminary recurrence-time calibration and model refinement

The published faricimab time to fourth injection was treated as a visit-observed endpoint rather than the unobserved biological recurrence time. Naive Weibull and log-logistic survival functions were first fitted directly to the reported injection-free survival probabilities. A schedule-adjusted Weibull model then generated a continuous recurrence time and assigned detection to the first eligible review. The first post-loading review was sampled from a normal distribution with mean 4 weeks and standard deviation 1 week, truncated to 2-8 weeks; later review increments had mean 6 weeks and standard deviation 1.5 weeks, truncated to 3-10 weeks. Shape and scale were estimated on the log scale by Nelder-Mead minimisation of mean squared error across 100,000 common-random-number trajectories. Fit was compared using root mean squared error.

The preliminary aflibercept 8 mg model reproduced the PULSAR year-1 dose-regimen-modification schedule and included a Bernoulli post-injection dry state, a latent Weibull reactivation time and the probability that active disease met the protocol modification rule. Calibration used 25,000 simulated eyes and final target evaluation used 100,000 eyes. Log- and logit-transformed parameters were estimated by multistart Nelder-Mead minimisation of scaled squared deviations from week-16 fluid control, week-48 interval maintenance, injection counts and week-48 fluid control, with weak regularisation against implausible parameter values.

Longitudinal versions of the recurrence model were assessed against PULSAR week-96 and TENAYA/LUCERNE week-112 interval, injection, BCVA and retinal-thickness targets. Calibration used 30,000 simulated eyes and final appraisal used 100,000 eyes. Although numerical optimisation converged, the homogeneous recurrence model did not reproduce held-out interval transitions, placed severe activity at a parameter boundary and did not identify a credible retinal-thickness rebound term. The aggregate sources also lacked the eye-level transition frequencies, recurrent-event histories and joint fluid–BCVA–retreatment data required to identify a Markov or multistate model. A more complex dynamic model would therefore have depended on additional untestable structural assumptions. The persistent Q8W/Q12W/Q16W mixture was retained as a parsimonious summary of dominant interval requirement; it was not interpreted as implying that clinical intervals never change. None of the rejected model’s comparative visual or anatomical predictions was retained.

Supplementary Appendix S4. Latent interval-capability model and evidence synthesis

The final treatment-burden model assigned each eye a persistent maximum interval capability k in {Q8W, Q12W, Q16W}. For evidence source s, the capability vector p_s followed a Dirichlet distribution. When class counts were observed, p_s was assigned Dirichlet(1+n_8, 1+n_12, 1+n_16). For the schedule-adjusted faricimab durability source, the class probabilities were 1-S(8), S(8)-S(12) and S(12), where S(t) was the fitted Weibull survival function; these probabilities were converted to a Dirichlet distribution using an effective sample size of 100. This prevented the 827-eye reinjection cohort from being treated as 827 direct interval assignments.

The other faricimab source distributions used Dirichlet parameters 1+(4,6,15) for the direct three-dose cohort, 1+(22,45,69) for the Australian completer cohort and 1+(134,211,286) for pooled TENAYA/LUCERNE. For aflibercept 8 mg, the PULSAR vector (Q8W,Q12W,Q16W)=(0.13,0.09,0.78) was represented as Dirichlet[1+291 x (0.13,0.09,0.78)]. In the Swiss real-world sensitivity source, 43.9% were assigned Q8W and the remaining 56.1% were divided between Q12W and Q16W using the conditional PULSAR ratio among eyes reaching at least Q12W; an effective sample size of 91 was used. Intervals beyond Q16W were assigned to Q16W because the common protocol was capped at Q16W.

Thirty thousand draws were generated from each source distribution. Fixed source weights were specified and frozen before the final comparative simulations. They were relevance weights rather than posterior probabilities, risk-of-bias scores or probabilities that a source was correct. Assignment considered similarity to the treatment-naïve target population, compatibility with the three-dose loading and Q8W-to-Q16W framework, directness of the interval/durability endpoint, study design, follow-up duration, completeness of interval reporting and potential cohort overlap. Faricimab weights were 0.408 for schedule-adjusted three-dose durability, 0.143 for the direct Doi cohort, 0.245 for Australian real-world evidence and 0.204 for pooled TENAYA/LUCERNE. Aflibercept 8 mg weights were 0.741 for PULSAR and 0.259 for Swiss real-world evidence. The source-specific rationale is

summarised in Supplementary Table S3. Narrow and broad weight distributions, source-pair analyses and leave-one-source-out analyses examined uncertainty around these transportability judgements.

Supplementary Appendix S5. Common-protocol estimands and treatment-burden calculations

The common schedule generated fixed class-specific injection totals n_k(t). Through week 52, the Q8W, Q12W and Q16W classes received 8, 7 and 7 injections; through week 104, they received 15, 11 and 10 injections. For treatment d and horizon t, expected injections were calculated as E[N_d(t)] = sum_k p_dk n_k(t). Expected visits were calculated in the same way after adding the week-12 non-injection assessment. The treatment contrast was Delta(t) = E[N_A8(t)] - E[N_FAR(t)]; negative values indicate fewer injections with aflibercept 8 mg.

The primary estimand was the difference in cumulative injections at weeks 52 and 104 under the common protocol, using interval-capability distributions derived from treatment-naïve sources in which mean visual outcomes were maintained under the reported regimens. No new eye-level BCVA threshold was imposed because the primary model did not simulate longitudinal visual trajectories. The conditioning referred to source-level maintenance of mean visual outcomes and external BCVA plausibility checks. Secondary summaries were expected visits, capability probabilities, the probability that either treatment required fewer injections, the probability of equal burden and the probabilities that the absolute contrast was below 0.5 or 1.0 injection. These cut-offs were operational summaries and were not treated as validated minimal clinically important differences.

Supplementary Appendix S6. Evidence-source uncertainty and influence analyses

Primary estimates used the fixed relevance weights. Weight uncertainty was examined by drawing w from a Dirichlet distribution centred on the fixed weight vector w_0, with concentration 40 for the narrow analysis and 8 for the broad analysis. The sampler generated one gamma-variate column for each Dirichlet parameter. Run-stopping audits required the mean simulated weights to agree with w_0 within 0.005 for the narrow analysis and 0.010 for the broad analysis (Supplementary Figure S1).

Five source-pair specifications were considered: the evidence-weighted synthesis, a pivotal-trial-based comparison, a direct three-dose faricimab comparison against PULSAR, a paired real-world comparison and an early faricimab durability comparison. Leave-one-source-out analyses removed one source at a time, renormalised the remaining weights and repeated the 104-week calculation. Treatment-burden superiority was not claimed when clinically credible specifications changed the direction of the contrast (Supplementary Figure S2).

Supplementary Appendix S7. Paired virtual-eye microsimulation and Monte Carlo assessment

Treatment-specific marginal capability vectors were coupled with a Gaussian copula. For each paired eye, correlated standard-normal variables (Z_FAR,Z_A8) with correlation rho were converted to uniform ranks by the standard-normal cumulative distribution function and then assigned to capability classes by inverse-transform categorical sampling. The primary correlation was rho=0.50; rho=0 and rho=1.00 were sensitivity settings. This correlation was not estimated from clinical data. Marginal mean burden does not depend on rho, whereas eye-level probabilities of fewer, equal or greater injections do. The distribution of paired 104-week injection differences in the primary cohort is shown in Supplementary Figure S3, and the corresponding joint treatment-specific capability matrix at rho=0.50 is shown in Supplementary Figure S4.

The primary cohort contained 100,000 counterfactually paired virtual eyes drawn from the fixed evidence-weighted marginals. Each record contained the capability class under each treatment, injection and visit counts through weeks 52 and 104, final interval and paired injection difference. Treatment schedules were fixed conditional on capability. An event-level sample of all injection weeks was retained for 1,000 paired eyes. The scenario grid used 100,000 pairs for each of five evidence specifications at rho=0, 0.50 and 1.00, giving 1.5 million paired evaluations. Common random numbers were used across source specifications within each correlation setting.

The nested probabilistic analysis selected 5,000 outer capability-parameter draws by deterministic stratification across the 30,000-draw analytic burden distribution. Within each outer draw, 1,000 independent antithetic correlated pairs were simulated, giving five million paired evaluations. The class-probability-weighted expectation was calculated exactly for each outer draw and used for the parameter uncertainty interval; inner simulations were used for paired-eye probabilities and Monte Carlo diagnostics. Monte Carlo standard error was SD(Delta_eye)/sqrt(M), where M was the number of paired eyes.

Simulation appraisal required all six marginal capability probabilities to be within 0.006 of their targets, the primary and nested 104-week mean contrasts to be within 0.035 injection of the frozen analytic target, the mean nested simulation error to be no greater than 0.015 injection in absolute value, and the spread of the mean contrast across correlation settings to be no greater than 0.060 injection for every source specification and horizon. The observed appraisal results are reported in Supplementary Table S4.

Supplementary Appendix S8. Exploratory longitudinal aggregate-data models

The exploratory archive contained 24 reports grouped into 18 study families, yielding 100 continuous-outcome rows and 38 standardised fluid rows. The analyses were restricted to treatment-naïve cohorts and to observations through week 60 for the week-16 and week-52 models. Year-2 values were retained as external descriptive anchors because aflibercept 8 mg long-term evidence was dominated by the single PULSAR family. The study registry and outcome-level extraction were organised by study family to minimise double-counting from sequential or potentially overlapping reports.

For BCVA, the outcome y_ijt was change from baseline in ETDRS or ETDRS-equivalent letters for report i, time j and study family f. The multilevel model was y_ijt = beta_0 + beta_1 A8_i + beta_2 log(1+t/4) + beta_3[A8_i x log(1+t/4)] + beta_4(B_i-B_bar) + beta_5 L4_i + u_f + e_ijt, where A8_i indicated aflibercept 8 mg, B_i was baseline BCVA on the common ETDRS-equivalent scale, L4_i indicated four-dose loading and u_f was a random study-family intercept. Within the same study-family arm, Cov(e_ijt,e_iju) was set to rho x SE_ijt x SE_iju; correlations between different families were zero. Restricted maximum likelihood was used. The primary rho was 0.60, with 0.30 and 0.90 examined separately.

Retinal-thickness change used the same time, treatment, interaction, baseline and loading terms. The strict model accepted only central subfield thickness or central retinal thickness and required at least three independent families for each treatment. Because one aflibercept 8 mg family had a usable CST/CRT variance, this model could not be estimated under the prespecified threshold. The expanded sensitivity model included CST, CRT, central macular thickness, foveal thickness, central foveal thickness and mean retinal thickness, with measurement definition entered as an additional categorical moderator. Its predictions were labelled CST-equivalent and were presented as exploratory sensitivity estimates (Supplementary Figure S5).

IRF absence, SRF absence and complete retinal dryness were analysed separately from events and denominators. Logit-transformed proportions were fitted with treatment, log(1+t/4), their interaction, a four-dose-loading indicator and a random study-family intercept. A 0.5 continuity correction was applied only when a zero cell required it. At least three independent study families per treatment were required. IRF- and SRF-specific models did not meet this threshold; complete dryness was estimated from three families per treatment. Lesion inactivity and other non-equivalent fluid definitions were not combined with complete retinal dryness. Comparative week-104 fluid modelling was not undertaken (Supplementary Figure S6).

Treatment-specific predictions and contrasts at weeks 16 and 52 were obtained from 20,000 draws from the multivariate-normal distribution of the fitted coefficients. For the prespecified week-52 BCVA and expanded retinal-thickness contrasts, nominal two-sided Wald t tests were calculated from the exact fitted contrast and model-based standard error using the t reference distribution from the fitted meta-regression (23 degrees of freedom for BCVA and 30 for retinal thickness). Additional P values were not calculated for the sensitivity analyses, which were assessed using effect estimates, uncertainty intervals and directional consistency. Leave-one-family-out models, three-dose-only analyses, study-design restrictions, scale restrictions and the within-eye correlation analyses examined sensitivity to modelling choices. CR2 small-sample-adjusted cluster covariance estimates were calculated when the number of study families permitted. Held-out faricimab cohorts were compared with study-level predictive intervals that included coefficient uncertainty, between-family heterogeneity and the external study

sampling variance. The principal week-52 longitudinal contrasts and sensitivity analyses are summarised in Supplementary Table S2.

Supplementary Appendix S9. Evidence-appraisal rules and inferential conventions

For the longitudinal extension, evidence appraisal considered convergence, design-matrix rank, the number of independent families per treatment, sensitivity to the assumed within-eye correlation and loading regimen, leave-one-family-out influence and external prediction. A week-52 shift greater than 2 ETDRS letters for BCVA or 20 micrometres for retinal thickness was considered material. Results were described as meeting the prespecified exploratory checks when all criteria were satisfied; supportive but dependent on the available evidence base when a key source or scale analysis could not be completed; restricted to sensitivity analysis when broader outcome definitions were required; variable across sensitivity analyses when a clinically relevant analysis changed direction or magnitude; and descriptive when the minimum evidence threshold was not met. Machine-readable status codes corresponding to these categories were retained only in the computational outputs.

Point estimates from probabilistic analyses were reported as Monte Carlo means and 95% uncertainty intervals from the 2.5th and 97.5th percentiles. The exploratory meta-regressions used parameter or study-level predictive intervals as stated. No null-hypothesis P value was derived from the number of virtual eyes. Nominal two-sided model-based P values were reported only for the prespecified week-52 BCVA and expanded retinal-thickness contrasts. No confirmatory family of hypothesis tests was defined, and these secondary P values were not adjusted for multiplicity. They were interpreted descriptively alongside effect estimates, uncertainty intervals and sensitivity analyses. Directional probabilities and threshold probabilities were descriptive. All treatment contrasts were defined as aflibercept 8 mg minus faricimab unless stated otherwise.

Supplementary Appendix S10. Software and computational implementation.

Analyses were performed in R version 4.6.1 (2026-06-24 ucrt) on Windows 10 x64. The treatment-burden evidence synthesis and paired virtual-eye simulation used version-controlled base-R functions from stats, graphics, grDevices and utils. The longitudinal extension used metafor 5.0.1 for multilevel meta-regression and logit-proportion models, clubSandwich 0.7.0 for CR2 small-sample-adjusted cluster covariance analyses, mvtnorm 1.4.2 for multivariate-normal coefficient simulation and Matrix 1.7-5 for positive-definite covariance repair. Random-number seeds were fixed before each analysis. A consolidated summary of the statistical models, computational specifications and model-appraisal rules is provided in Supplementary Table S1.

Supplementary Tables

Supplementary Table S1. Detailed statistical models, simulations and model-appraisal rules.

Stage	Statistical method/model	Computational specification	Targets or decision rule
Supporting loading synthesis	Relevance-weighted inverse-total-variance pooling; logMAR-to-ETDRS conversion; reconstructed change-score SD; Beta-binomial updating	Source relevance divided by SE squared plus heterogeneity floor; r=0.50 for reconstructed change SD	Plausibility inputs only; no comparative efficacy claim
Faricimab durability	Naive Weibull, naive log-logistic and visit-schedule-adjusted Weibull survival models	100,000 common-random-number trajectories; Nelder-Mead estimation on log parameters	Model selected by RMSE; visit-observation process retained
Preliminary A8 calibration	Patient-level Weibull recurrence, Bernoulli dry state and probabilistic DRM detection	25,000 calibration eyes; 100,000 final eyes; multistart Nelder-Mead	Week-16/48 fluid, interval and injection targets
Longitudinal model appraisal	Source-specific PULSAR and TENAYA/LUCERNE interval algorithms with BCVA/CST bridge	30,000 calibration and 100,000 model-appraisal eyes	BCVA +/-2 letters; CST +/-20-25 micrometres; proportions +/-5%; injections +/-0.5; model not carried forward

Stage	Statistical method/model	Computational specification	Targets or decision rule
Final capability model	Persistent Q8W/Q12W/Q16W latent class mixture with Dirichlet source distributions	30,000 draws per source; weak Dirichlet(1,1,1) regularisation or effective sample sizes	Primary treatment-burden model
Evidence synthesis	Draw-wise convex combination using frozen source-relevance weights	FAR weights 0.408/0.143/0.245/0.204; A8 weights 0.741/0.259	Fixed-weight primary analysis
Weight uncertainty	Dirichlet uncertainty centred on frozen source weights	Concentrations 40 and 8; automated centre audits	Narrow and broad sensitivity analyses
Paired-eye probability framework	Gaussian copula with latent capability ranks	Applied within the 100,000-eye primary cohort and the scenario/correlation grid described below; rho=0, 0.50 and 1.00	P(A8 fewer), P(equal), P(FAR fewer)
Sensitivity and source influence	Five source-pair specifications and leave-one-source-out reanalysis	Weights renormalised after each omission	No superiority claim when credible specifications change direction
Primary paired virtual-eye cohort	Gaussian-copula paired microsimulation; inverse-transform Q8W/Q12W/Q16W assignment	100,000 paired eyes; rho=0.50; deterministic common-protocol schedules	Marginal probabilities within 0.006; 104-week mean within 0.035 injection; MCSE reported
Scenario/correlation grid	Five evidence scenarios evaluated under alternative cross-treatment dependence	100,000 pairs per scenario at rho=0, 0.50 and 1.00; 1.5 million evaluations	Mean difference spread across rho no greater than 0.060 injection
Nested probabilistic analysis	Stratified outer parameter draws with independent antithetic paired-eye samples	5,000 outer draws x 1,000 pairs; exact outer expectations plus five million eye evaluations	Parameter UI from analytic expectations; mean simulation error no greater than 0.015 injection
Synthetic-sample inference	Empirical paired-eye probabilities and Monte Carlo standard errors	No null-hypothesis test based on virtual-eye count	Synthetic N used for precision only; no clinical P values
Exploratory BCVA longitudinal extension	Multilevel REML meta-regression of ETDRS or ETDRS-equivalent change with treatment-by-log-time interaction, baseline BCVA converted to a common ETDRS-equivalent scale and loading-regimen adjustment	Primary within-eye correlation 0.60; sensitivities 0.30/0.90; working within-family-arm covariance; random study-family intercept; 20,000 coefficient draws; CR2 small-sample-adjusted covariance and leave-one-family-out checks	Weeks 16 and 52; all prespecified evidence-appraisal criteria were not considered met when any treatment-family omission left fewer than three families
Exploratory anatomical/fluid extension	Strict CST/CRT gate; expanded measurement-definition-adjusted thickness sensitivity; separate logit models for IRF absence, SRF absence and complete dryness	Minimum three independent families per treatment; week-104 comparison prohibited; non-equivalent fluid definitions kept separate	Anatomical findings treated as sensitivity analyses when broader definitions were required; fluid endpoints presented as supportive or descriptive according to the available family counts

Supplementary Table S2. Exploratory longitudinal contrasts and principal sensitivity analyses.

Outcome/analysis	Week-52 contrast (A8-FAR); model-based P where tested	95% UI	Status/implication
BCVA primary, r=0.60	+0.64 letter; t = 0.602; df = 23; P = 0.553	-1.41 to 2.70	Small and uncertain
BCVA, r=0.30	+0.74 letter	-1.91 to 3.40	Direction similar; assessed from estimate and uncertainty interval
BCVA, r=0.90	-0.85 letter	-3.60 to 1.90	Direction differed from the primary analysis; interval includes no difference
BCVA, three-dose only	+0.37 letter	-3.42 to 4.06	No material difference; sensitivity estimate only
BCVA source omission	A8 families not omittable	Not estimated under the prespecified threshold	Supports an exploratory interpretation in the context of the available evidence base
Thickness expanded model, r=0.60	+24.8 micrometres; t = 1.077; df = 30; P = 0.290	-20.7 to 69.2	Expanded-definition sensitivity analysis
Thickness, r=0.90	+4.7 micrometres	-56.5 to 65.4	Attenuated; sensitivity estimate only
Thickness, three-dose only	-27.0 micrometres	-72.6 to 18.4	Direction differed from the main analysis; between-treatment contrast treated as hypothesis-generating
Strict CST/CRT model	Not estimated under the prespecified threshold	Not estimated under the prespecified threshold	One A8 family with usable variance; result presented descriptively
IRF/SRF absence	Not estimated under the prespecified threshold	Not estimated under the prespecified threshold	Presented descriptively
Complete retinal dryness	Marginal probabilities only	No comparative contrast interval	Three families per treatment; presented as supportive exploratory evidence

Note: Model-based P values are reported only for the prespecified week-52 BCVA and expanded retinal-thickness contrasts. They were obtained from two-sided Wald t tests using the exact fitted contrasts, model-based standard errors and the t reference distribution from the fitted meta-regression. They were not adjusted for multiplicity and were not used to support confirmatory treatment claims. Additional P values were not calculated for sensitivity analyses, the virtual-eye sample or descriptive fluid-probability differences.

Supplementary Table S3. Prespecified evidence-relevance weights and transportability rationale.

Evidence source	Target-protocol directness	Design and endpoint features	Fixed weight	Rationale
Faricimab three-dose durability	High for loading; indirect conversion to interval class	Large treatment-naïve real-world reinjection cohort; time to fourth injection	0.408	Most direct three-dose durability source; effective sample size limited to avoid over-weighting.
Doi three-dose cohort	High regimen directness	Direct Q8W/Q12W/Q16W allocation; small, non-randomised and centre-confounded	0.143	Direct but imprecise interval evidence.
Australian faricimab real-world evidence	Moderate-high	Treatment-naïve registry; achieved intervals in routine practice	0.245	Pragmatic external evidence with clinical-pathway heterogeneity.
TENAYA/LUCERNE	Lower loading directness; high population similarity	Phase 3 randomised trials; four-dose loading and protocol-assigned intervals	0.204	High internal validity but less direct match to the common loading regimen.
PULSAR	High	Phase 3 randomised trial; three-dose loading and reported interval distribution	0.741	Principal aflibercept 8 mg source with the closest regimen match.
Swiss aflibercept 8 mg real-world evidence	Moderate	Treatment-naïve multicentre cohort; real-world interval attainment	0.259	Pragmatic source complementing PULSAR; healthcare-pathway and reporting differences remain.

Note: weights were frozen before the final comparative simulations and represent relevance to the target population, regimen and endpoint. They are not formal quality scores or posterior probabilities that a source is correct.

Supplementary Table S4. Simulation implementation and Monte Carlo appraisal results

Appraisal check	Target/reference	Simulated result	Appraisal statistic	Criterion	Outcome
Faricimab capability margins	0.257535/0.244676/0.497790	0.255240/0.246030/0.498730	Maximum absolute deviation 0.002295	≤0.006	Met
Aflibercept 8 mg capability margins	0.210766/0.085829/0.703406	0.211570/0.086570/0.701860	Maximum absolute deviation 0.001546	≤0.006	Met
Primary week-104 mean contrast	−0.392693	−0.377810	Absolute deviation 0.014883	≤0.035	Met
Nested analytic week-104 contrast	−0.392693 frozen target	−0.392671 selected-draw analytic result	Absolute deviation 0.000023	≤0.035	Met
Nested inner simulation, week 104	−0.392671 analytic	−0.393036 simulated	Mean signed error −0.000365; absolute error 0.000365	≤0.015	Met
Correlation-setting invariance	Analytic maximum spread 0.000	Empirical maximum spread 0.019590	0.019590 injection	≤0.060	Met
Primary Monte Carlo standard error	—	MCSE 0.001582 at week 52 and 0.007460 at week 104	Quantifies finite-simulation error	No predefined acceptance threshold	Not applicable

Note: Capability vectors are ordered Q8W/Q12W/Q16W. Appraisal statistics were calculated from unrounded values; therefore, some displayed differences may not equal differences calculated from the rounded values shown.

Supplementary Figures

Supplementary Figure S1. Evidence-weight uncertainty around the fixed 104-week source weights. Mean injection differences (aflibercept 8 mg minus faricimab) were -0.39 (95% UI -0.68 to -0.11) with fixed weights, -0.39 (-0.77 to -0.01) under the narrow distribution and -0.39 (-0.94 to 0.23) under the broad distribution. The narrow and broad analyses used Dirichlet concentration parameters of 40 and 8, respectively.

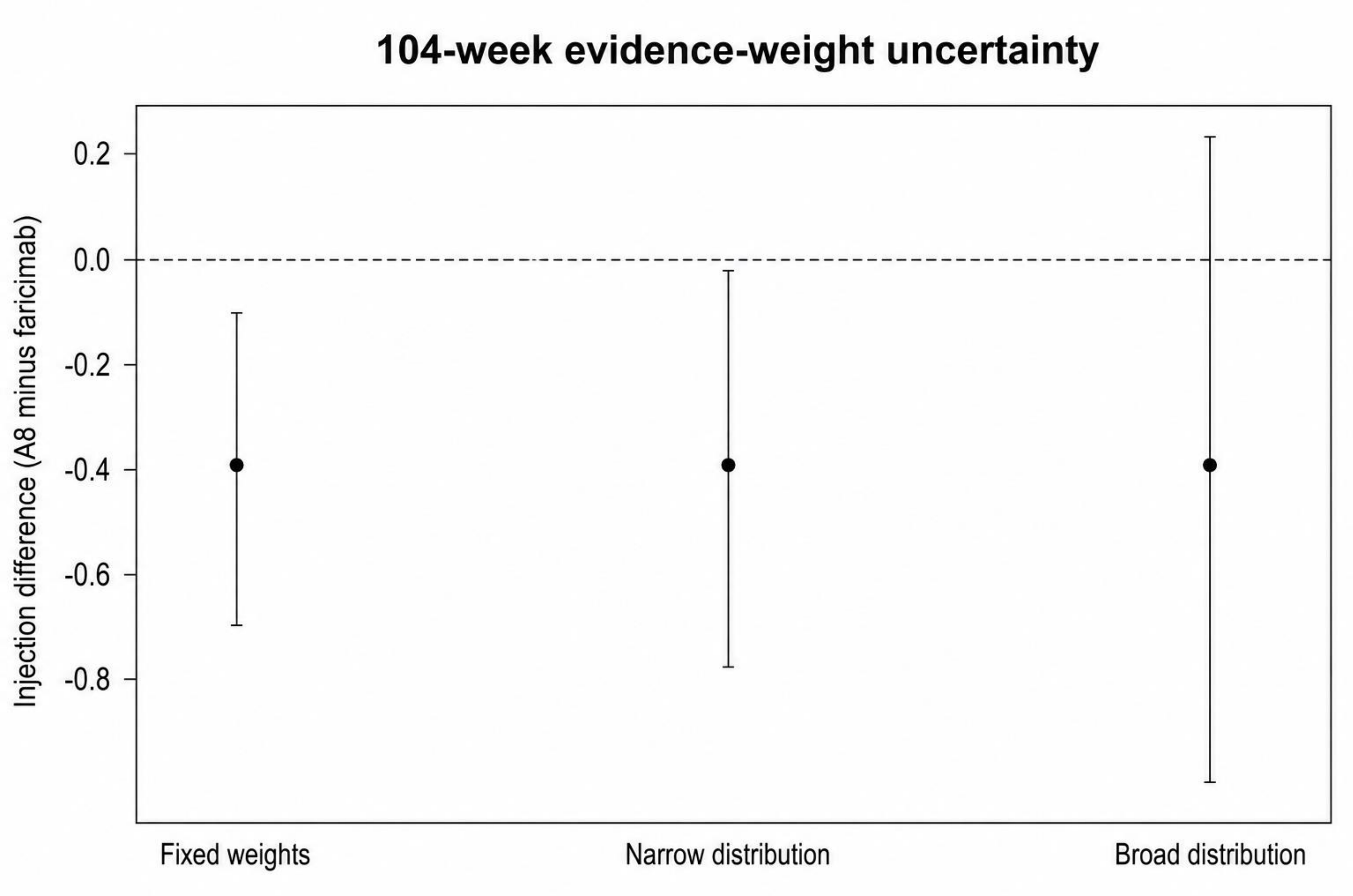

Supplementary Figure S2. Leave-one-source-out influence analysis at week 104. The mean differences (95% UI) after omitting each source were: three-dose faricimab durability, -0.10 (-0.38 to 0.18); Doi, -0.46 (-0.76 to -0.16); Australian faricimab real-world evidence, -0.51 (-0.85 to -0.19); TENAYA/LUCERNE, -0.43 (-0.76 to -0.10); PULSAR, +0.71 (0.19 to 1.24); and Swiss aflibercept 8 mg real-world evidence, -0.78 (-1.07 to -0.49). Negative values favour aflibercept 8 mg; positive values favour faricimab.

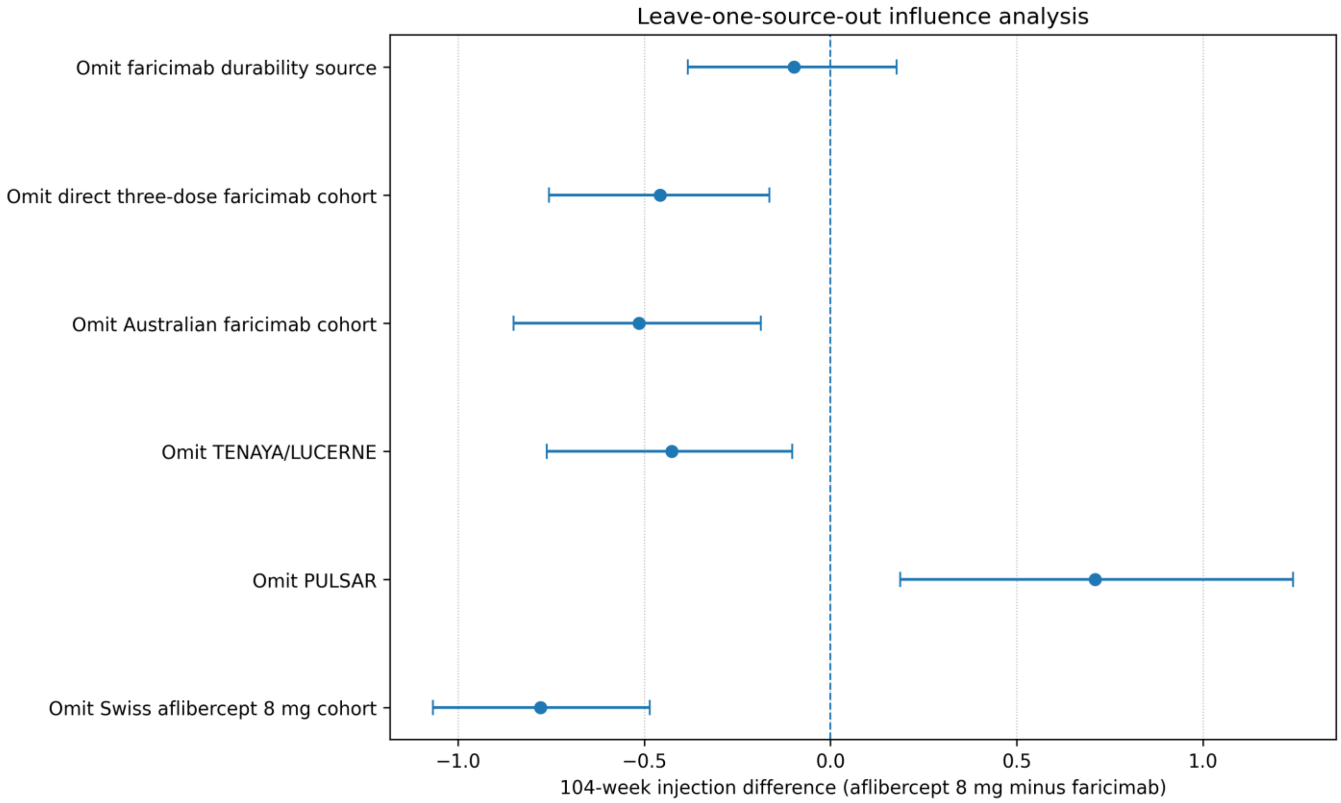

Supplementary Figure S3. Distribution of the paired 104-week injection difference in the primary virtual-eye cohort, defined as aflibercept 8 mg minus faricimab. Aflibercept 8 mg required fewer injections in 31.15% of paired virtual eyes, burden was equal in 55.41% and faricimab required fewer injections in 13.44%.

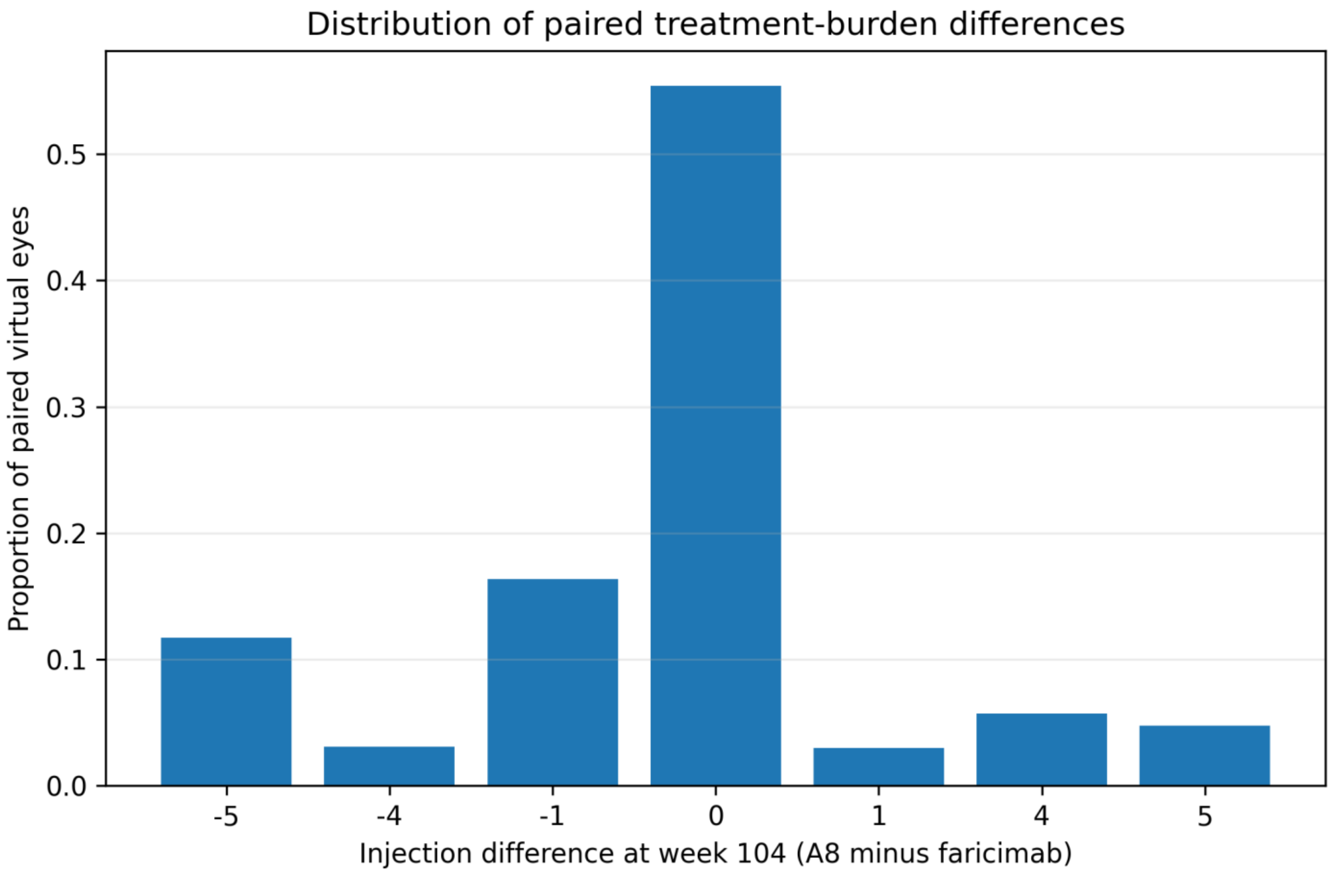

Supplementary Figure S4. Joint treatment-specific interval-capability distribution in the primary cohort at a Gaussian-copula rank correlation of 0.50. The marginal Q8W/Q12W/Q16W proportions were 25.52%/24.60%/49.87% for faricimab and 21.16%/8.66%/70.19% for aflibercept 8 mg; cells show the percentage assigned to each joint capability combination.

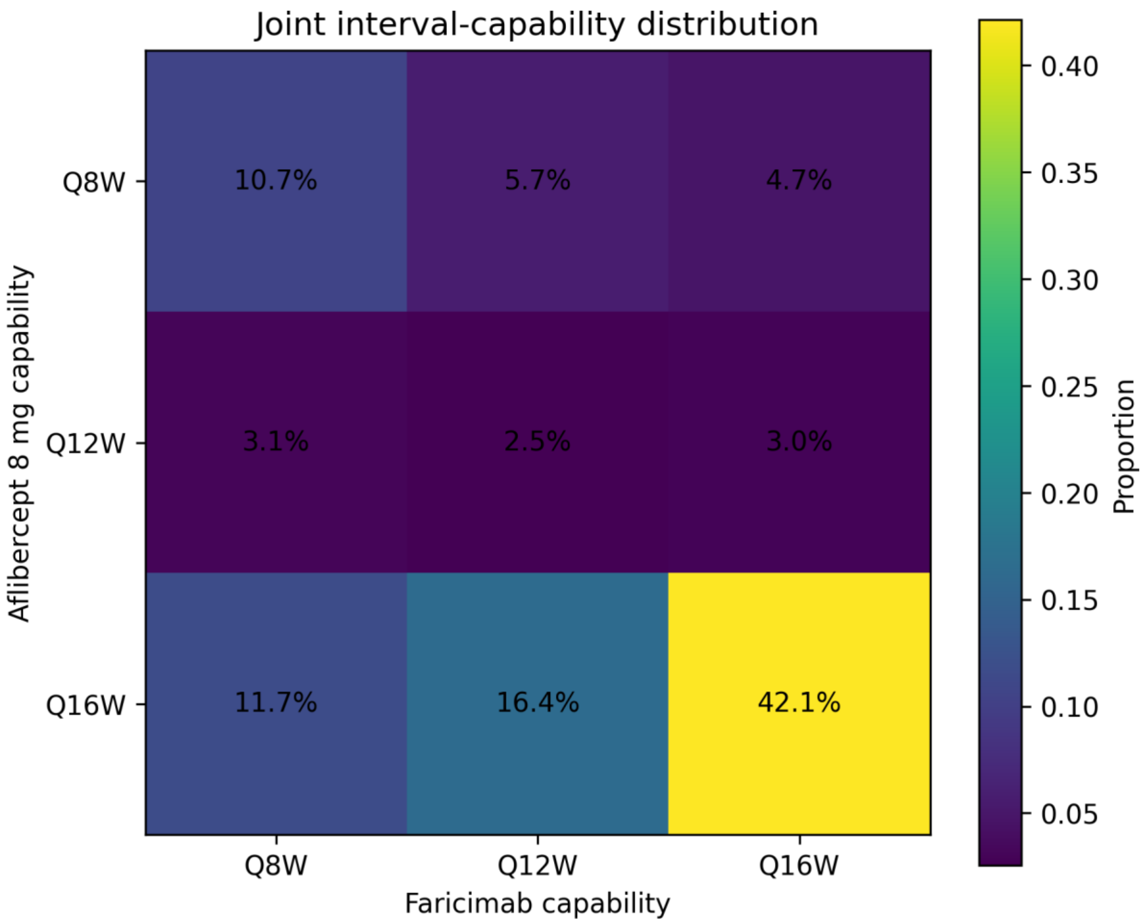

Supplementary Figure S5. Model-based CST-equivalent retinal-thickness trajectories from the expanded measurement-definition sensitivity analysis. At week 16, projected changes were -160.9 micrometres (95% UI -192.5 to -129.5) with faricimab and -142.7 micrometres (-191.2 to -94.4) with aflibercept 8 mg. At week 52, the corresponding changes were -171.8 micrometres (-202.6 to -141.1) and -147.2 micrometres (-195.9 to -98.4). Uncertainty curves are omitted from the figure for readability and the numerical intervals are reported here.

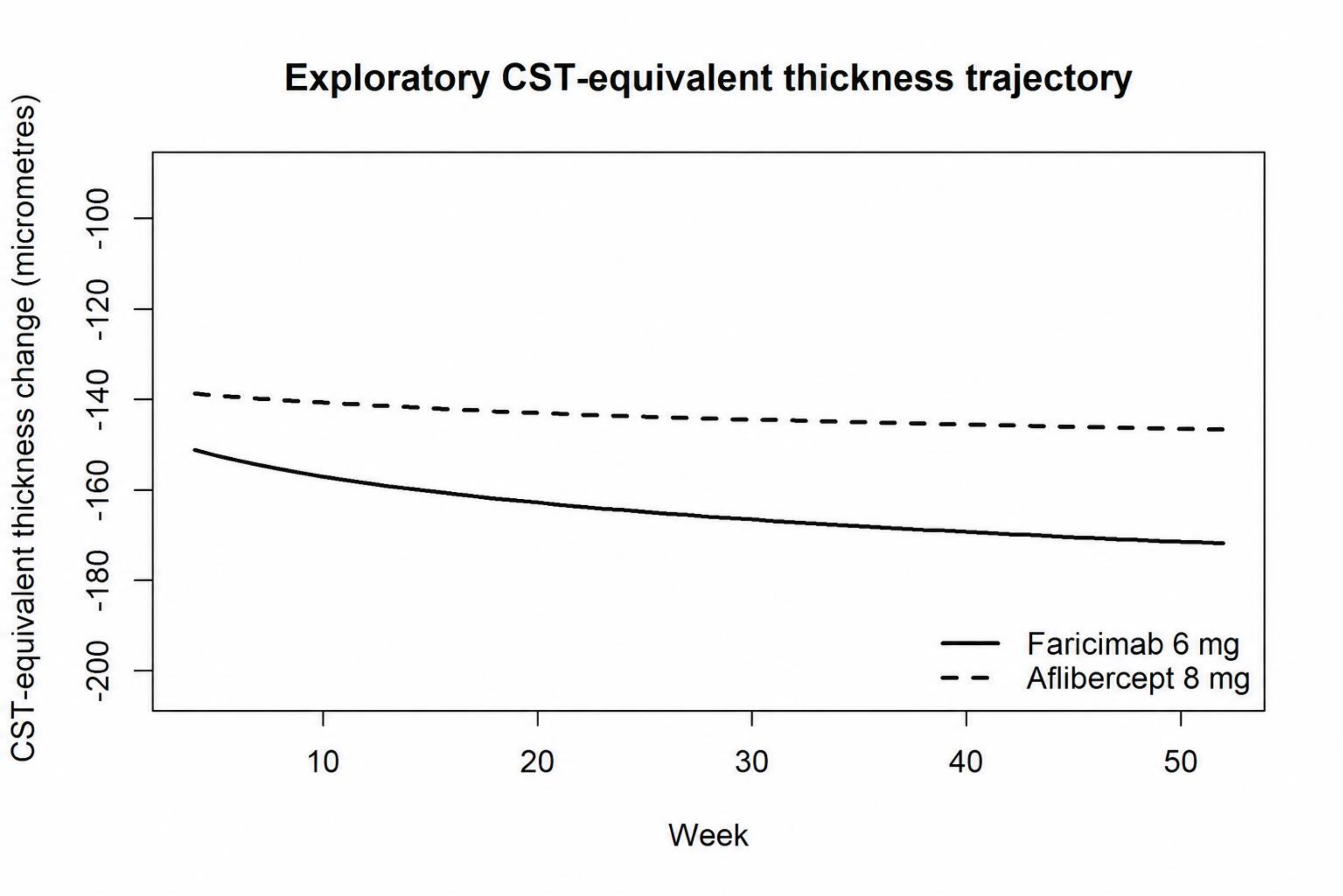

Supplementary Figure S6. Exploratory complete retinal-dryness probabilities. At week 16, projected probabilities were 45.9% (95% UI 39.1%-52.8%) with faricimab and 64.1% (60.6%-67.5%) with aflibercept 8 mg. At week 52, they were 57.0% (48.6%-65.1%) and 69.0% (65.0%-72.7%), respectively. The endpoint included three study families per treatment and is presented as supportive exploratory evidence, not as comparative anatomical superiority.

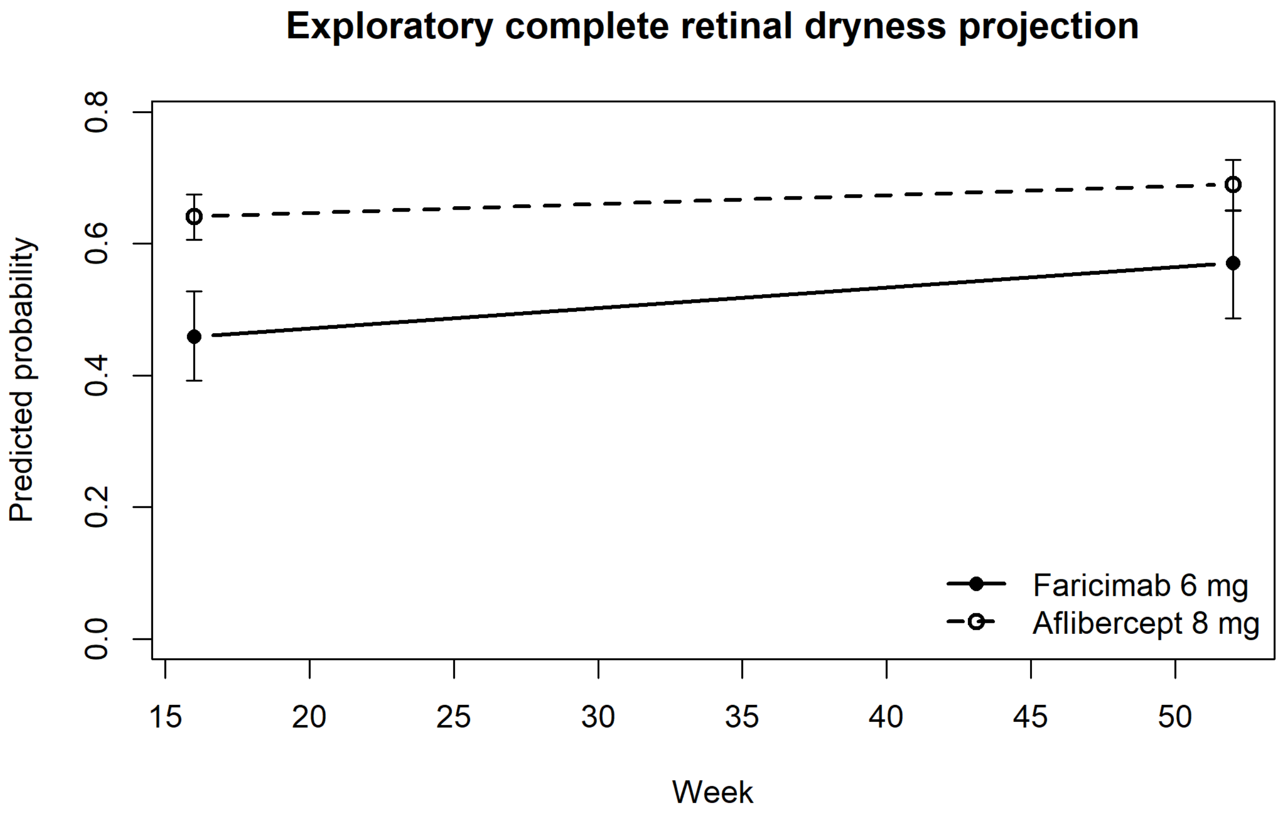

Supplement: Supplementary file 1 [file pharmaceuticals-19-01316-s001.zip › pharmaceuticals-4476957-supplementary.pdf]
